# Fumarate Hydratase Lactylation Exacerbates Traumatic Brain Injury Pathology via Mitochondrial Dysfunction and Neuroinflammation

**DOI:** 10.1002/advs.77698

**Published:** 2026-09-27

**Authors:** Yang Tian, Shuoyao Ma, Bo Yang, Fei Gao, Hao Guo, Haixiao Liu, Xun Wu, Wenxing Cui, Jingyu Dong, Qian Lin, Yufeng Ge, Jin Ma, Jiazhen Zhao, Yongzhi Zhang, Dongzhi Xue, Jiaxin Tian, Ziwen Zhang, Kai Wang, Meng Xu, Dayun Feng, Jinpeng Zhou, Yan Qu

**Affiliations:** ^1^ Department of Neurosurgery Tangdu Hospital Fourth Military Medical University Xi'an China; ^2^ Department of Cardiology Xijing Hospital Fourth Military Medical University Xi'an China; ^3^ Department of Critical Care Medicine Air Force 986 Hospital Fourth Military Medical University Xi'an China; ^4^ Department of Neurosurgery First Affiliated Hospital of Harbin Medical University Harbin China; ^5^ Shaanxi Provincial Neurosurgical Medical Quality Control Center Xi'an China; ^6^ Shaanxi Provincial Brain Injury Assessment Quality Control Center Xi'an China

**Keywords:** cerebral edema, fumarate hydratase, lactylation, mitochondrial dysfunction, neuroinflammation, traumatic brain injury

## Abstract

Traumatic brain injury (TBI) induces brain tissue ischemia, hypoxia, and heightened glycolysis, leading to lactate accumulation. Lactate exerts multiple pathophysiological effects in the aftermath of TBI. This study aimed to elucidate the role of lactate‐mediated lactylation in these post‐TBI pathological processes. Here, we found that the level of lactylation was elevated in brain tissues after TBI and predominantly in neurons. Proteomic analysis revealed that mitochondrial proteins underwent significant lactylation. Lactylation at the K112 site of fumarate hydratase (FH) induced mitochondrial damage and tricarboxylic acid (TCA) cycle dysfunction. Accumulation of fumarate and release of mitochondrial DNA (mtDNA) activated the cGAS‐STING pathway of the innate immune response, thereby exacerbating neuroinflammation and cerebral edema. Furthermore, AARS2 and SIRT3 were identified as the respective “writer” and “eraser” of FH K112 lactylation. The short peptide Pep‐K112 targeted and inhibited lactylation at the FH K112 site, ameliorating mitochondrial function and alleviating neuroinflammation and cerebral edema. These findings demonstrate the regulatory role of lactylation in the pathological progression of TBI and suggest that targeting FH lactylation may be a promising strategy for TBI treatment.

## Graphical abstract

Tian et al. reveal that FH K112 lactylation is elevated after TBI, leading to fumarate accumulation, mitochondrial damage, mtDNA release and amplified neuroinflammation. AARS2 and SIRT3 act as the “writer” and “eraser” for FH K112 lactylation. The peptide Pep‐K112 targeting this site alleviates brain injury, highlighting a potential therapeutic target.

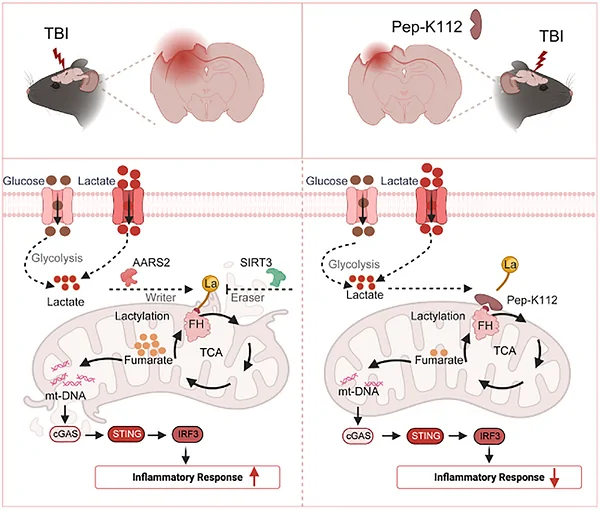

## 1 Introduction

Traumatic brain injury (TBI) is a leading cause of death and disability worldwide, with an annual incidence exceeding 50 million [1]. Secondary brain injury, a major contributor to high disability and mortality rates after TBI, induces cerebral edema, thereby triggering irreversible neuronal injury, cerebral herniation, and even death [2, 3, 4]. However, current treatments for secondary brain injury remain limited to symptomatic supportive therapies, and no targeted therapies are available [3, 5]. Therefore, elucidating the mechanisms underlying secondary brain injury after TBI constitutes a critical and urgent research priority.

Insufficient microcirculatory perfusion in brain tissue following TBI suppresses oxidative phosphorylation and enhances glycolysis, leading to massive lactate accumulation [6, 7]. Lactate is not only an energy metabolite but also a critical precursor for protein lactylation [8]. As a novel post‐translational modification (PTM), lactylation participates in gene expression, metabolism, and immune responses, thus playing a vital regulatory role in acute brain injury [9].

Although initial investigations primarily focused on histone lactylation in transcriptional regulation [10], emerging evidence demonstrates that non‐histone lactylation regulates distinct pathological axes with cell‐type and disease specificity [11, 12]. Lactylation of the mitochondrial elongation factor Tufm following TBI inhibits mitophagy, leading to the accumulation of damaged mitochondria and subsequent neuronal apoptosis [13]. In astrocytes, ARF1 lactylation impairs its capacity to transfer mitochondria to neurons, exacerbating secondary brain injury after TBI [14]. Conversely, in ischemic stroke, lactylation of the transcriptional regulator MeCP2 enhances its binding to pro‐apoptotic gene promoters, suppressing their transcription and reducing neuronal death to confer neuroprotection [11]. Additional protective non‐histone lactylation events occur post‐ischemia, indicating that non‐histone lactylation orchestrates a complex regulatory network in acute brain injury, where site‐specific modifications on distinct proteins may determine cell fate [15]. However, the mechanisms by which non‐histone lactylation regulates neuroinflammation and cerebral edema following TBI remain unclear, and targeted interventions specifically against individual modification sites are still lacking.

In this study, we demonstrate that lactylation of mitochondrial fumarate hydratase (FH) at lysine 112 (K112) is significantly upregulated following TBI. This modification specifically impairs FH enzymatic activity, resulting in fumarate accumulation, which disrupts mitochondrial membrane potential and triggers mtDNA release. The released mtDNA subsequently activates the cGAS‐STING pathway, exacerbating neuroinflammation and cerebral edema. Mechanistically, we identify AARS2 and SIRT3 as the respective “writer” and “eraser” enzymes for FH K112 lactylation. Furthermore, intervention with the FH K112‐targeted peptide Pep‐K112 effectively attenuated cerebral edema and neuropathological damage in TBI mice. These findings highlight FH lactylation as a critical driver of TBI pathogenesis and a promising therapeutic target.

## 2 Results

### 2.1 Lactate Accumulation After TBI Promotes Lactylation

**FIGURE 1:**
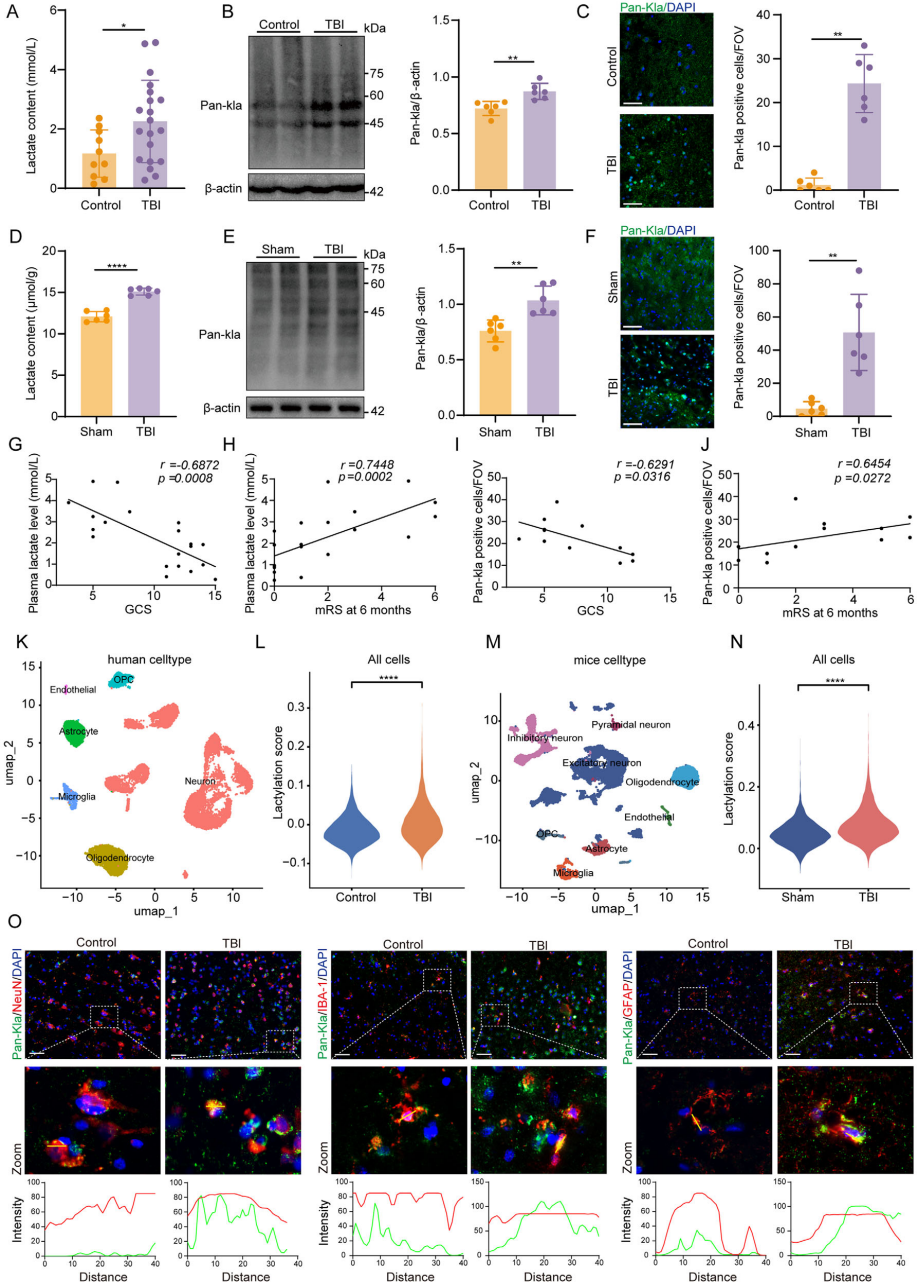
Lactate accumulation after TBI promotes lactylation. (A) Plasma lactate levels in control (n = 10) and TBI (n = 20) patients upon admission. (B) Western blot analysis of Pan‐Kla expression in brain tissues of control and TBI patients (n = 6 per group). (C) Representative immunofluorescence images of Pan‐Kla in control and TBI patients (n = 6 per group). Scale bar, 50 µm. (D) Lactate content of brain tissue in sham and TBI mice at 24 h after injury (n = 6 per group). (E) Western blotting analysis of Pan‐Kla expression in brain tissues of sham and TBI mice at 24 h after injury (n = 6 per group). (F) Representative immunofluorescence images of Pan‐Kla in sham and TBI mice at 24 h after injury (n = 6 per group). Scale bar, 50 µm. (G) Pearson correlation analysis between plasma lactate levels and GCS score at admission (n = 20). (H) Pearson correlation analysis between plasma lactate levels at admission and mRS score at 6 months after discharge (n = 20). (I) Pearson correlation analysis between Pan‐Kla expression assessed by immunofluorescence and GCS score at admission (n = 12). (J) Pearson correlation analysis between Pan‐Kla expression assessed by immunofluorescence and mRS score at 6 months post‐discharge (n = 12). (K) UMAP visualization of brain cells from control and TBI patients. (L) Global lactylation scores of all cell types from snRNA‐seq data in control and TBI patients. (M) UMAP visualization of brain cells from sham and TBI mice. (N) Global lactylation scores of all cell types from snRNA‐seq data in sham and TBI mice. (O) Representative immunofluorescence images showing co‐localization of Pan‐Kla with neuron (NeuN), microglia (IBA‐1), astrocyte (GFAP) in brain tissue from control and TBI patients. Scale bar, 50 µm. Data are presented as mean ± SD. *p < 0.05, **p < 0.01, ****p < 0.0001. Two‐tailed unpaired Student's t test (A‐F). Pearson correlation analysis (G‐J). Two‐sided unpaired Wilcoxon test (L, N).

To evaluate lactate metabolism and lactylation in brain edema tissue after TBI, we measured plasma lactate concentrations in TBI patients and controls at admission, and also assessed lactylation levels in surgically resected brain edema tissue samples. Patient information is provided in Tables S1–S3. The results indicated that lactate concentrations and lactylation levels were increased in patients following TBI (Figure 1A–C). Consistently, both lactate levels and lactylation in edematous brain tissues were significantly upregulated in mice after TBI (Figure 1D–F). Additionally, both plasma lactate levels and Pan‐Kla immunofluorescence result in brain edema tissue of TBI patients were negatively correlated with admission Glasgow Coma Scale (GCS) scores and positively correlated with modified Rankin Scale (mRS) scores at 6 months after discharge (Figure 1G–J). These results are consistent with previous studies, suggesting that lactate and lactate‐mediated lactylation are key factors contributing to poor prognosis after TBI [13, 16, 17].

To further elucidate the dynamics of lactylation after TBI, we performed analysis on single‐nucleus RNA sequencing (snRNA‐seq) data derived from TBI patient in the GEO database (GSE209552) [18], which revealed 6 distinct cell types (Figure 1K and Figure S1A). Analysis of lactylation scores across all cell types revealed a significant increase in global lactylation after TBI (Figure 1L). Separate scoring of lactylation demonstrated a marked elevation in each cell type after TBI (Figure S1B). In addition, snRNA‐seq of brain edema tissue from mice at 1 day after TBI identified 8 distinct cell types (Figure 1M and Figure S1C). Notably, the lactylation scores in TBI mice were consistent with human (Figure 1N and Figure S1D). To identify the primary cell types exhibiting lactylation after TBI, we performed immunofluorescence staining of Pan‐Kla on edematous brain tissue samples obtained from patients and mice after TBI. The results confirmed that lactylation predominantly occurs in neurons after TBI (Figure 1O and Figure S1E).

### 2.2 Mitochondrial Protein Lactylation is Elevated After TBI

**FIGURE 2:**
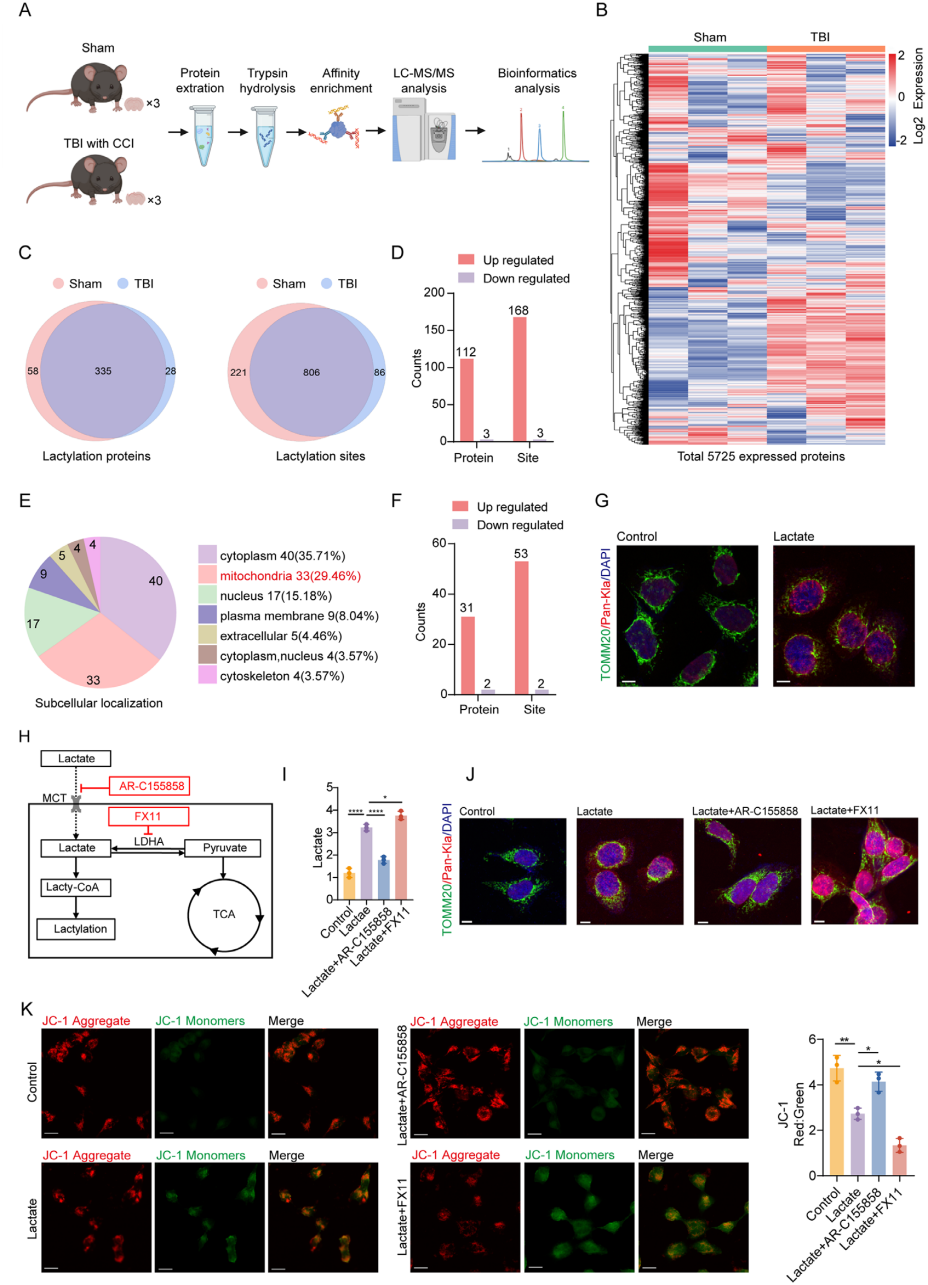
Mitochondrial protein lactylation is elevated after TBI. (A) Flow chart illustrating LC‐MS/MS and bioinformatic analysis of sham and TBI mice at 24 h post‐injury. (B) Heatmap displaying the expression profiles of 5,725 proteins in sham and TBI mice. (C) Venn diagram illustrating shared and specific lactylated proteins and lactylation sites in sham and TBI mice. (D) The number of differentially expressed lactylated proteins and lactylation sites after TBI. (E) The subcellular localization of differentially lactylated proteins. Percentages are rounded to two decimal places. (F) The number of differentially expressed lactylated proteins and lactylation sites in mitochondria after TBI. (G) Representative immunofluorescence images of Pan‐Kla co‐stained with TOMM20 in control and lactate groups. Scale bar, 10 µm. (H) Schematic diagram of experimental intervention approaches in vitro (MCT inhibitor AR‐C155858, 1 µM, LDHA inhibitor FX11, 9 µM. The bidirectional arrow denotes biochemical reversibility, not net flux direction). (I) Lactate levels in indicated groups of HT22 cells (n = 3 independent experiments). (J) Representative immunofluorescence images of Pan‐Kla co‐stained with TOMM20 in different groups. Scale bar, 10 µm. (K) Representative images and analysis of mitochondrial JC‐1 staining in different groups. Scale bar, 20 µm (n = 3 independent experiments). Data are presented as mean ± SD. *p < 0.05, **p < 0.01. One‐way ANOVA followed by Bonferroni post hoc test (I and K).

To characterize the proteomic features of lactylation during the acute phase after TBI, we performed proteomic sequencing on edematous mouse brain tissue at 1 day after TBI to assess protein lactylation levels (Figure 2A). A total of 5,725 proteins were identified (Figure 2B), among which 393 lactylated proteins and 1,027 lactylation sites were detected in the sham group, and 363 lactylated proteins and 892 lactylation sites were identified in the TBI group (Figure 2C). Additionally, 335 lactylated proteins and 806 lactylation sites were shared between the sham group and the TBI group (Figure 2C). Compared to the sham group, TBI induced statistically significant changes in 115 lactylated proteins and 171 lactylation sites (Fold change > 1.5 or Fold change < 0.67, and *p* value < 0.05) (Figure 2D).

Subcellular localization analysis revealed that following TBI, differentially lactylated proteins predominantly localized to the cytoplasm (35.71%) and mitochondria (29.46%) (Figure 2E). A total of 33 differentially lactylated proteins and 55 differentially modified lactylation sites were identified on mitochondria after TBI (Figure 2F). Immunofluorescence staining in HT22 cells confirmed that mitochondrial protein lactylation increased following lactate intervention (Figure 2G). Western blot analysis of proteins from edematous brain tissues of humans and mice further confirmed that the level of mitochondrial protein lactylation was elevated after TBI (Figure S2A,B).

To elucidate the functional role of lactylation in mitochondria, we applied inhibitors against core regulators of lactate transport and metabolism (Figure 2H). Following exogenous lactate intervention, mitochondrial protein lactylation increased, accompanied by decreased membrane potential and fragmented morphology. When the monocarboxylate transporter (MCT) was inhibited by ARC‐155858, mitochondrial lactylation was decreased, membrane potential recovered, and mitochondrial integrity improved. Furthermore, inhibition of lactate dehydrogenase A (LDHA) with FX11 increased intracellular lactate levels and mitochondrial protein lactylation, which further reduced mitochondrial membrane potential and compromised mitochondrial structural integrity (Figure 2I–K and Figure S2C,D). These results demonstrate that lactate enhances mitochondrial protein lactylation, leading to dysfunction and disruptions in mitochondrial morphological integrity.

### 2.3 FH is Lactylated at K112 After TBI

**FIGURE 3:**
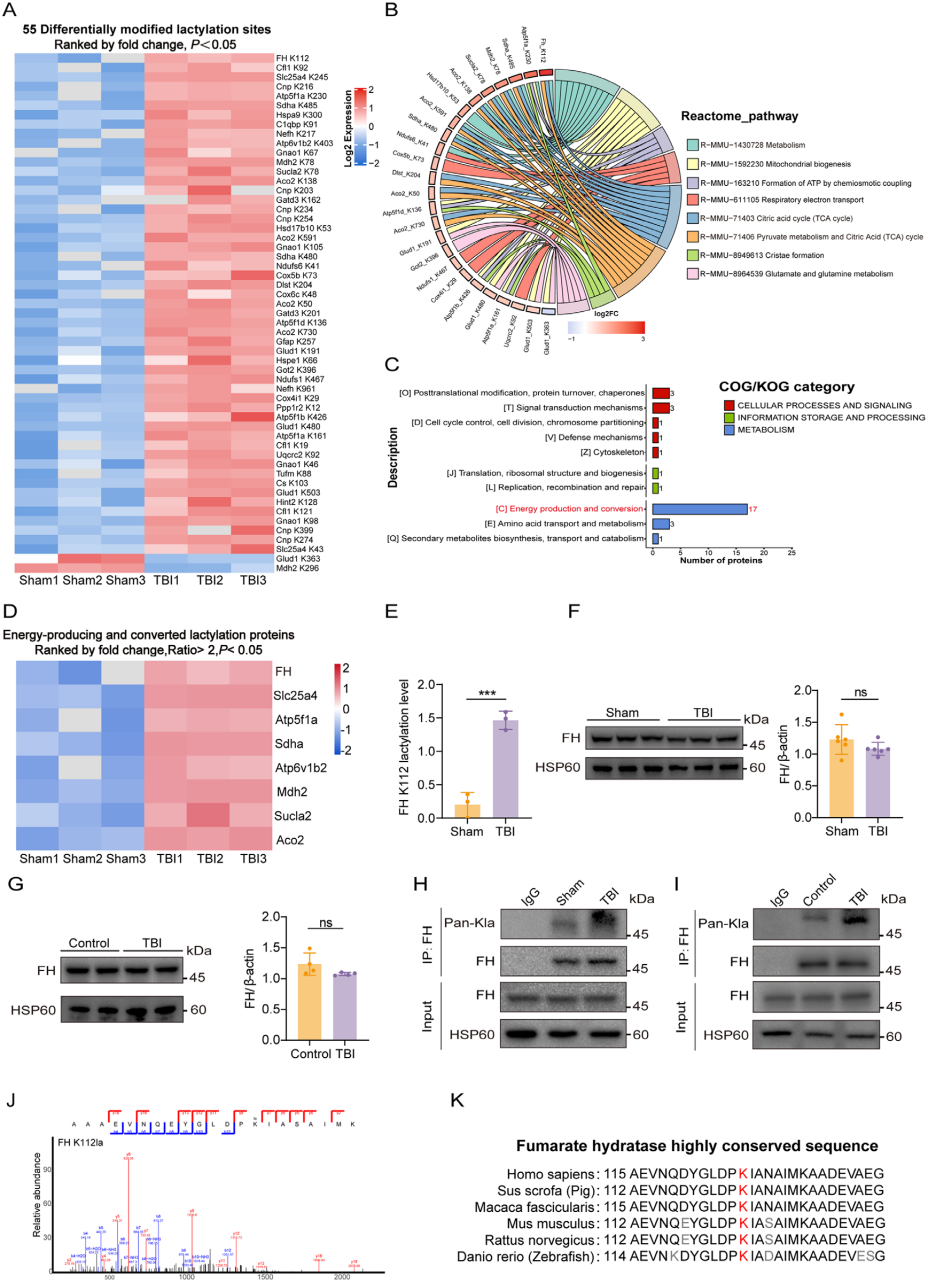
FH is lactylated at K112 after TBI. (A) Heatmap illustrating the ranking of differentially lactylated sites in mitochondria of mice after TBI. (B) Reactome enrichment analysis of mitochondrial protein lactylation sites in mice after TBI. (C) COG/KOG functional annotation of proteins with differentially lactylated sites in mitochondria. (D) Heatmap displaying the top 8 differentially lactylated proteins involved in energy production and conversion. (E) Relative quantification of lactylation at the FH K112 site via MS/MS (n = 3 per group). (F) Western blot analysis of FH expression in sham and TBI mice (n = 6 per group). (G) Western blot analysis of FH expression in control and TBI patients (n = 4 per group). (H) Tissue lysates were immunoprecipitated with FH antibody, followed by immunoblotting with Pan‐Kla antibody to assess FH lactylation levels in sham and TBI mice. (I) Tissue lysates were immunoprecipitated with FH antibody, followed by immunoblotting with Pan‐Kla antibody to assess FH lactylation levels in control and TBI patients. (J) Illustration of FH K112 site lactylation identified by MS. (K) Sequence alignment of FH proteins across different species. Data are presented as mean ± SD. ***p < 0.001, ns, not significant. Two‐tailed unpaired Student's t‐test (E‐G).

Given that mitochondria serve as the central hub of cellular energy metabolism and regulate cell fate through metabolic integration and innate immune activation [19, 20, 21], we further investigated mitochondrial lactylated proteins after TBI. The heatmap visualizes 55 lactylation modification sites across all 33 differentially modified mitochondrial proteins (Figure 3A). Reactome pathway analysis indicated that the functions of proteins corresponding to differentially lactylated sites in mitochondria were mainly concentrated in metabolism and the tricarboxylic acid (TCA) cycle (Figure 3B and Figure S2E). COG/KOG analysis indicated that lactylated mitochondrial proteins were predominantly enriched in metabolic pathways, particularly energy production and conversion (Figure 3C). Notably, KEGG functional classification and Wiki pathway analyses consistently corroborated these findings (Figure S2F,G). Collectively, these findings indicate that lactylation may likely compromise mitochondrial protein functionality in energy metabolism pathways, leading to and functional impairment of mitochondria.

We further analyzed the mitochondrial lactylated proteins involved in energy production and conversion, and screened the top 8 mitochondrial proteins with the most significant lactylation modification (Fold change ratio >2, *p* < 0.05) (Figure 3D). The mRNA levels of these proteins were inconsistent with their protein expression levels. This discrepancy suggests that translation or PTM may have potential impacts on protein expression (Figure S3A,B). Importantly, FH exhibited the most significant lactylation modification on mitochondria following TBI (Figure 3D,E). To further investigate the effect of TBI on FH expression levels, we performed protein immunoblotting on edematous brain tissues from humans and mice after TBI. Both samples consistently indicated no significant alteration in FH protein expression (Figure 3F,G). Additionally, immunohistochemical staining of human edematous brain tissues supported this conclusion (Figure S3C) by confirming that lactylation of FH does not alter its protein expression levels after TBI.

Subsequently, we assessed lactylation levels of FH after TBI. Endogenous FH from human and mouse edematous brain tissues was immunoprecipitated using an FH antibody, followed by assessment of FH lactylation via Pan‐Kla immunoblotting. The results revealed that lactylation levels of FH were significantly elevated after TBI (Figure 3H,I). We utilized Deep‐Kla to predict lysine residues on FH potentially subject to lactylation modification [22]. The machine learning platform predicted 6 lysine sites with high modification probability (>60%) (Figure S3D). Among these, only the K112 lysine residue was experimentally validated via immunoprecipitation coupled to liquid chromatography‐tandem mass spectrometry (LC‐MS/MS) (Figure 3J). Notably, this site exhibits high conservation across different species (Figure 3K). These results identify K112 as the key site for FH lactylation after TBI. To characterize the correlation between lactate and FH lactylation, we quantified their levels at serial time points post‐TBI. Lactate elevation preceded progressive FH lactylation, which peaked at 24 h before declining, revealing lactate accumulation as a trigger for FH lactylation (Figure S3F‐G).

### 2.4 FH K112 Lactylation Impairs Mitochondrial Function and Triggers mtDNA Release

**FIGURE 4:**
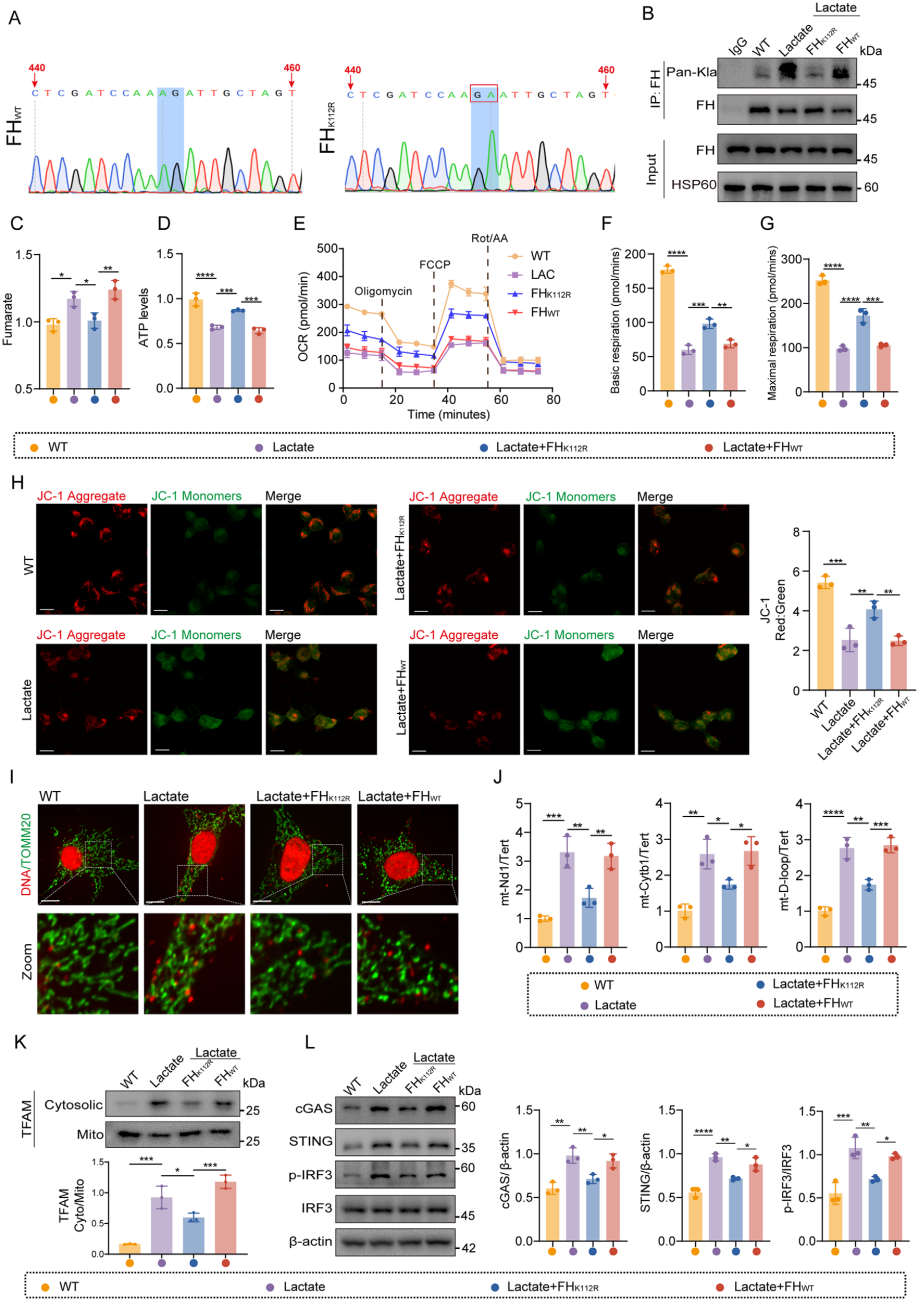
FH K112 lactylation impairs mitochondrial function and triggers mtDNA release. (A) Lactylation‐mimetic lysine mutants. Blue‐shaded lines and red boxes indicate lysine mutation sites. (B) Western blot analysis of FH lactylation levels in HT22 cells transfected with FH_WT_ and FH_K112R_ plasmids following lactate treatment. (C) Fumarate levels in HT22 cells expressing FH_WT_ or FH_K112R_ plasmids after lactate treatment (n = 3 independent experiments). (D) ATP levels of HT22 cells expressing FH_WT_ or FH_K112R_ plasmids after lactate treatment (n = 3 independent experiments). (E) OCR measurements during the MitoStress test in HT22 cells in indicated groups (n = 3 independent experiments). (F) Quantification of basal respiratory capacity in indicated groups (n = 3 independent experiments). (G) Quantification of maximal respiratory capacity in indicated groups (n = 3 independent experiments). (H) Representative images and quantitative analysis of mitochondrial JC‐1 staining in indicated groups. Scale bar, 20 µm (n = 3 independent experiments). (I) Representative immunofluorescence images of mtDNA co‐stained with TOMM20 in indicated groups. Scale bar, 5 µm. (J) qPCR quantification of cytosolic mtDNA (mt‐Nd1, mt‐Cytb, mt‐D‐loop) in HT22 cells, normalized to nuclear DNA (Tert) from whole‐cell lysates (n = 3 independent experiments). (K) Western blot analysis of TFAM expression in the cytosol and mitochondria in indicated groups (n = 3 independent experiments). (L) Western blot and quantification of cGAS, STING, and p‐IRF3 protein expression in different groups (n = 3 independent experiments). Data are presented as mean ± SD. *P< 0.05, **p < 0.01, ***p < 0.001, ****p < 0.0001. One‐way ANOVA followed by Bonferroni post hoc test (C‐D, F‐H, and J‐L).

As a key enzyme in the TCA cycle and a critical component of the mitochondrial oxidative respiratory chain, FH is primarily responsible for catalyzing the conversion of fumarate to malate, energy production, and maintenance of mitochondrial complex activity [23, 24, 25]. We performed molecular docking simulations to assess alterations in the binding energy between FH and fumarate before and after K112 lactylation. The binding energy changed from −4.4 kcal/mol to −4.1 kcal/mol upon K112 lactylation, suggesting that this modification weakens substrate‐binding affinity and may contribute to reduced FH catalytic activity (Figure S4A). To evaluate the impact of FH lactylation at the K112 site on the aforementioned functions, we mutated the K112 residue of FH to arginine and detected its lactylation level as well as the effects on mitochondria in HT22 cells treated with lactate (Figure 4A). The FH K112R mutation inhibited the exogenous lactate‐mediated increase in FH lactylation (Figure 4B). Accordingly, the FH K112R mutation effectively reversed the intracellular fumarate accumulation triggered by exogenous lactate (Figure 4C). In terms of mitochondrial energy generation, FH K112R restored lactate‐induced adenosine triphosphate (ATP) depletion (Figure 4D). Seahorse assays further revealed that lactate suppressed cellular respiration, whereas intervention via FH K112R mutation significantly restored mitochondrial respiratory capacity (Figure 4E–G). Notably, mitochondrial complex activity measurements revealed that FH K112R reversed the lactate‐induced reduction in complex activity (Figure S4B). Further analysis of mitochondrial membrane potential and morphology confirmed that FH K112R intervention alleviated lactate‐mediated mitochondrial dysfunction and compromised morphological integrity (Figure 4H and Figure S4C,D).

Previous studies have reported that excessive fumarate accumulation induces mitochondrial morphological abnormalities and structural disruption, impairs mitochondrial structural stability, and promotes aberrant mtDNA release into the cytosol via sorting nexin 9 (SNX9)‐dependent budding and trafficking of mitochondria‐derived vesicles (MDVs) from the mitochondrial membrane [26, 27, 28]. Therefore, we measured the cytosolic mtDNA content in vitro. Immunofluorescence staining indicated increased mitochondrial mtDNA leakage after lactate intervention, whereas FH K112R treatment reversed this leakage (Figure 4I). We quantified cytosolic mtDNA using three mtDNA‐specific primers (mt‐ND1, mt‐Cytb and mt‐D‐loop), and the results were in agreement with the immunofluorescence findings (Figure 4J). Given that mitochondrial transcription factor A (TFAM) is the primary binding protein for leaked mtDNA in the cytosol [29], we further quantified TFAM expression in both cytosolic and mitochondrial fractions. Results showed that lactate intervention increased cytosolic TFAM levels, whereas FH K112R treatment effectively inhibited this lactate‐induced increase (Figure 4K). These results reveal FH K112 lactylation as a key event triggering mitochondrial damage and fumarate accumulation, which consequently promote mtDNA leakage into the cytosol. As mtDNA is an integral component of the cGAS‐STING immune response, we examined key proteins in this pathway. Leakage of mtDNA led to increased expression of cGAS, STING, and p‐IRF3, while FH K112R counteracted these effects (Figure 4L). To investigate the SNX9 dependence of fumarate accumulation‐induced mtDNA release, we treated cells with an SNX9 inhibitor in vitro to evaluate the cytosolic mtDNA content. The results indicated that suppressing SNX9 reduced the leakage of mtDNA into the cytosol. These findings suggest that the elevated fumarate levels induced by FH lactylation trigger the aforementioned SNX9‐mediated mtDNA release process (Figure S4E,F).

### 2.5 AARS2 and SIRT3 Regulate FH K112 Lactylation as “Writer” and “Eraser”

**FIGURE 5:**
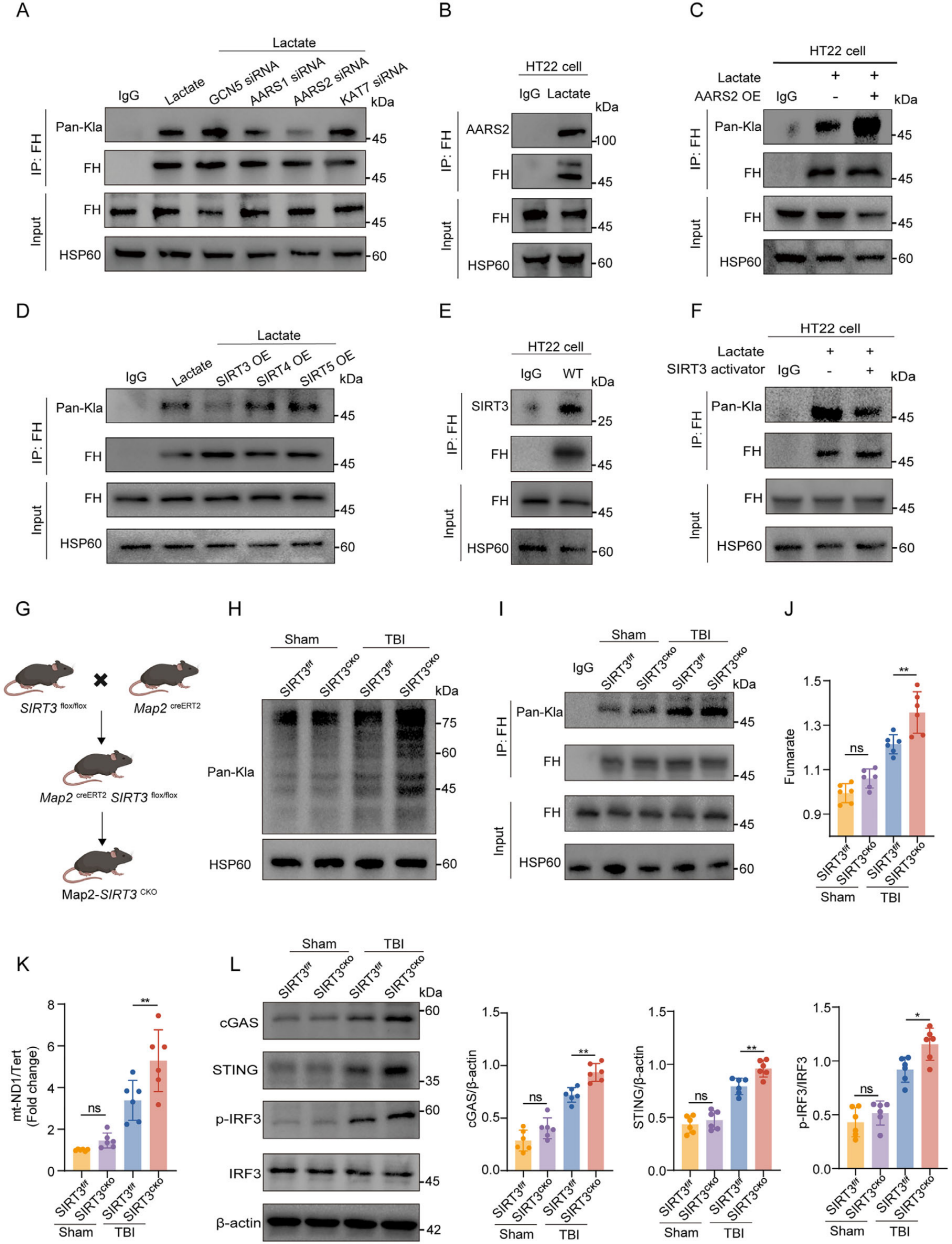
AARS2 and SIRT3 Regulate FH K112 Lactylation as “Writer” and “Eraser”. (A) Western blot analysis of FH lactylation levels in HT22 cells following knockdown of GCN5, AARS1, AARS2 or KAT7. (B) Co‐IP analysis of AARS2 in HT22 mitochondrial lysates. (C) Western blot analysis of FH lactylation levels in HT22 cells following overexpression of AARS2. (D) Western blot analysis of FH lactylation levels in each group of HT22 cells following overexpression of SIRT3, SIRT4 or SIRT5. (E) Co‐IP analysis of SIRT3 in HT22 mitochondrial lysates. (F) HT22 cells were treated with SIRT3 activator (5 µM), and cell mitochondrial lysates were immunoprecipitated with FH antibody, followed by detection of the FH lactylation levels. (G) Schematic diagram of the construction of SIRT3 conditional knockout mice. (H) Western blot analysis of Pan‐Kla lactylation levels in different groups of mice. (I) Western blot analysis of FH lactylation levels in different groups of mice. (J) Fumarate levels in the brain tissues of indicated groups of mice (n = 6 per group). (K) qPCR measurement of cytosolic mtDNA (mt‐Nd1) in indicated groups of mice (n = 6 per group). (L) Western blot analysis of cGAS, STING, and p‐IRF3 protein expression in different groups of mice (n = 6 per group). Data are presented as mean ± SD. *p < 0.05, **p < 0.01, ns, not significant. One‐way ANOVA followed by Bonferroni post hoc test (J‐L).

To elucidate the regulatory mechanism underlying FH K112 lactylation, we investigated the enzymes mediating this modification. IP‐MS analysis revealed four established lactyltransferases: AARS1, AARS2, KAT2 (GCN5), and KAT7 (HBO1) (Table S4). To determine which enzyme mediates FH lactylation, we performed siRNA‐mediated knockdown of each candidate (Figure 5A). We found that knockdown of AARS2 significantly attenuated FH lactylation levels (Figure 5A and Fig S5A). Co‐IP assays confirmed physical interaction between AARS2 and FH (Figure 5B and Figure S5B). Consistently, overexpression of AARS2 significantly promoted the lactylation level of FH (Figure 5C). Additionally, considering the potential roles of P300/CBP and GLO1 (associated with non‐enzymatic lactylation) in this process [30], we evaluated FH lactylation levels following inhibition of P300/CBP and GLO1 in cellular models. Our results indicated that inhibiting P300/CBP or GLO1 did not significantly affect FH lactylation (Figure S5C,D).

Given that Sirtuin family proteins are key delactylases and SIRT3‐5 are localized within mitochondria, we selected SIRT3, SIRT4 and SIRT5 as candidate enzymes for mitochondrial protein delactylation [31, 32, 33]. In vitro experiments confirmed that SIRT3 reduced the overall lactylation levels of mitochondrial proteins as well as the lactylation level of FH (Figure S5E,F and Figure 5D). We further verified SIRT3‐FH interaction by Co‐IP (Figure 5E, Figure S5G), consistent with their binding identified from IP‐MS (Table S4). Both in vivo and in vitro, intervention with a SIRT3 agonist reduced FH lactylation levels, whereas the SIRT3 inhibitor produced the opposite effect (Figure 5F and Figure S5H–K). Molecular docking results also indicated that lactylation at the K112 site decreased the binding energy of the FH‐SIRT3 docking (Figure S5L). Furthermore, downregulated SIRT3 expression after TBI (Figure S5M) represents one possible cause for increased FH lactylation upon TBI.

To further investigate the role of SIRT3 in FH lactylation in vivo, we generated neuron‐specific SIRT3 knockout mice by crossing SIRT3*^flox/flox^* mice with MAP2*^creERT2^* mice (Figure 5G and Figure S5N). Results showed that conditional knockout of SIRT3 significantly increased global lactylation of mitochondrial proteins after TBI (Figure 5H). Immunoprecipitation assays further confirmed elevated lactylation of FH (Figure 5I). Additionally, SIRT3 conditional knockout led to increased fumarate levels following TBI (Figure 5J). Next, we examined mtDNA content in brain edema tissue from TBI mice. Results showed that SIRT3 cKO led to significant upregulation of cytosolic mtDNA (Figure 5K and Figure S5O). Similarly, SIRT3 cKO markedly increased the expression of cGAS‐STING pathway‐related proteins (Figure 5L). Collectively, SIRT3 functions as the delactylating enzyme for FH. Down‐regulation of SIRT3 after TBI promotes FH hyperlactylation, leading to fumarate accumulation, mtDNA leakage, and subsequent cGAS‐STING pathway activation.

### 2.6 Pep‐K112 Inhibits FH K112 Lactylation

**FIGURE 6:**
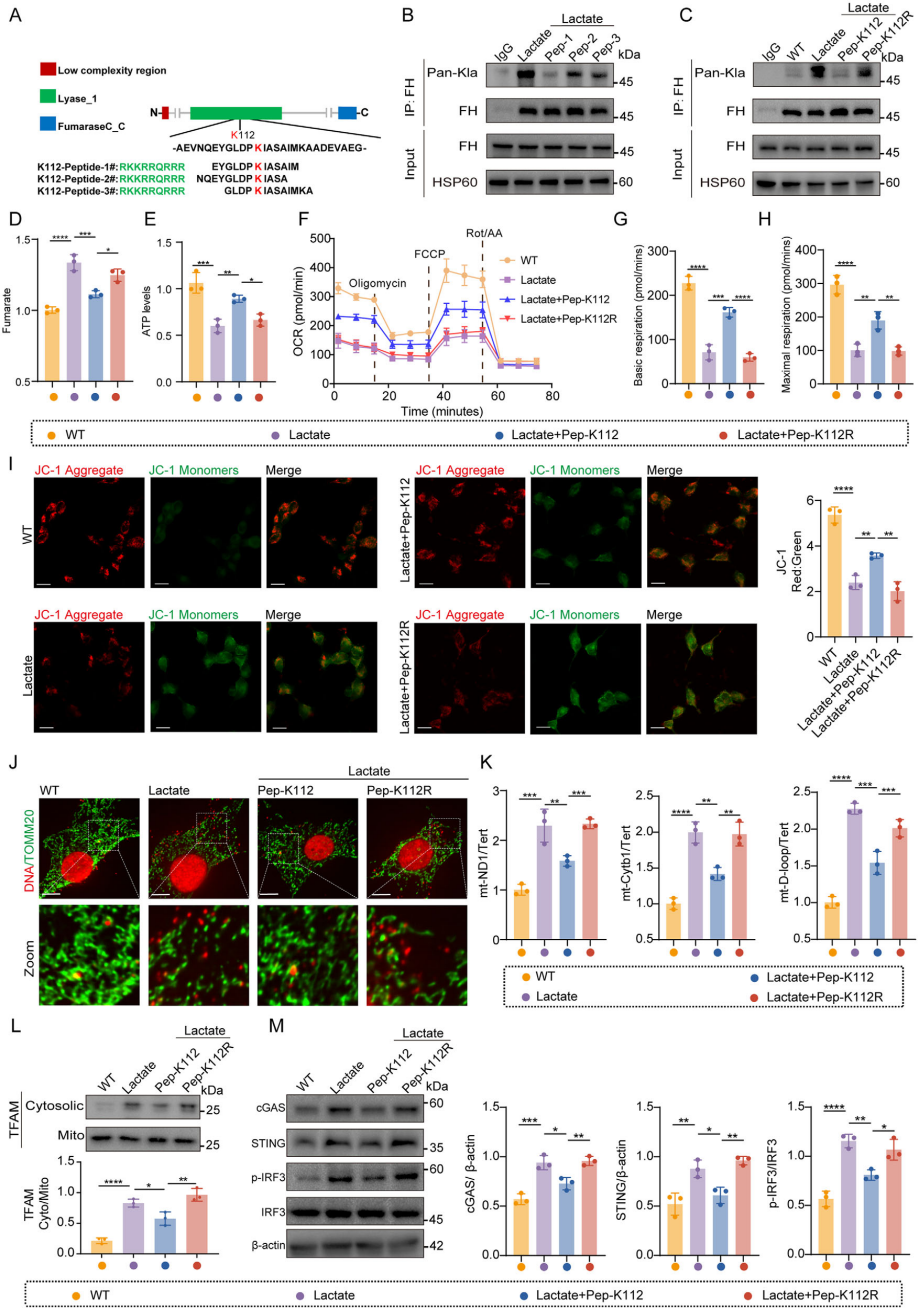
Pep‐K112 Inhibits the Lactylation of FH K112. (A) Schematic diagram of peptide design. Green represents the cell‐penetrating peptide (CPP), and red indicates the FH lactylation site. (B) Western blot analysis of FH lactylation levels after treatment with indicated peptides. (C) Western blot analysis of FH lactylation levels after treatment with Pep‐K112 or Pep‐K112R. (D) Fumarate levels in indicated groups of HT22 cells (n = 3 independent experiments). (E) ATP levels of HT22 cells in indicated groups (n = 3 independent experiments). (F) OCR measurements during the MitoStress test in HT22 cells from different groups. (G) Quantification of basal respiratory capacity in indicated groups (n = 3 independent experiments). (H) Quantification of maximal respiratory capacity in different groups (n = 3 independent experiments). (I) Representative images and quantitative analysis of mitochondrial JC‐1 staining in indicated groups. Scale bar, 20 µm (n = 3 independent experiments). (J) Representative immunofluorescence images of mtDNA co‐stained with TOMM20 in indicated groups. Scale bar, 5 µm. (K) qPCR quantification of cytosolic mtDNA (mt‐Nd1, mt‐Cytb, mt‐D‐loop) in HT22 cells, normalized to nuclear DNA (Tert) (n = 3 independent experiments). (L) Western blot analysis of TFAM expression in the cytosol and mitochondria of indicated groups (n = 3 independent experiments). (M) Western blot analysis of cGAS, STING, and p‐IRF3 expression in indicated groups (n = 3 independent experiments). Data are presented as mean ± SD. *p < 0.05, **p < 0.01, ***p < 0.001, ****p < 0.0001. One‐way ANOVA followed by Bonferroni post hoc test (D‐E, G‐I, and K‐M).

Peptides designed derived from PTM motifs have proven a promising and efficient approach for targeted PTM inhibition. After analyzing the sequence around the FH K112 site, we designed 3 TAT(RKKRRQRRR)‐conjugated peptides and validated their cell‐penetrating capacity and mitochondrial targeting in vitro (Figure 6A and Figure S6A). After confirming their safety via cell viability assays (Figure S6B), we evaluated their inhibitory effects on FH lactylation, with Pep‐1 exhibiting the most potent suppression (Figure 6B). To emphasize Pep‐1 sequence features, we renamed it Pep‐K112.

After verifying the optimal intervention concentration of Pep‐K112 in vitro (Figure S6C,D), we synthesized a mutant peptide (Pep‐K112R) based on the Pep‐K112 sequence for functional validation. Pep‐K112 suppressed FH lactylation and fumarate accumulation while elevating cellular ATP levels. In contrast, Pep‐K112R failed to elicit these beneficial effects (Figure 6C–E). Seahorse analysis further confirmed that Pep‐K112 rescued lactate‐mediated repression of cellular respiration, whereas Pep‐K112R exerted no comparable regulatory function (Figure 6F–H). Additionally, Pep‐K112 enhanced mitochondrial function and maintained morphological integrity, inhibited mtDNA leakage, and suppressed downstream cGAS‐STING pathway activation. In contrast, Pep‐K112R treatment afforded no mitochondrial protective effects (Figure 6I—L and Figure S6E–G). Collectively, these findings demonstrate that in vitro targeted inhibition of FH K112 lactylation by Pep‐K112 improves mitochondrial function and mitigates mtDNA leakage‐induced cGAS‐STING pathway activation.

To directly confirm the targeting capability of Pep‐K112, we designed two types of control peptides: an HA‐tagged peptide and an HA‐tagged K112‐mutant peptide. Following in vitro intervention, IP‐WB and Immunoprecipitation mass spectrometry (IP‐MS) were performed (Figure S6H). The results demonstrated that upon HA‐Pep‐K112 treatment, FH specifically captured the HA‐tagged peptide, whereas no such interaction was detected in the control groups. This confirms the highly specific targeting capability of Pep‐K112 toward FH (Figure S6I). To determine whether Pep‐K112 specifically targets the K112 site of FH without affecting other lysine residues, we generated an FH‐K112R mutant and treated cells with HA‐tagged Pep‐K112. The results showed that mutation of the K112 residue in FH abolished the capture of the HA‐tagged peptide, indicating that the interaction between Pep‐K112 and FH is dependent on the K112 site. These findings demonstrate that Pep‐K112 functions primarily through recognition of the K112 site of FH, rather than through non‐specific binding to other lysine residues on the protein (Figure S6J). To evaluate potential off‐target effects, we cross‐referenced the proteins identified by IP‐MS with our lactylome data. Notably, the Pep‐K112 peptide specifically enriched FH among the previously identified lactylated proteins, indicating no significant off‐target effects (Figure S6K). Finally, whole‐proteome analysis revealed that Pep‐K112 intervention reshaped the cellular metabolic and inflammatory landscape, with the upregulation of TCA cycle‐related proteins and a potent suppression of acute inflammatory response pathways (Figure S6L‐M).

### 2.7 Pep‐K112 Attenuates Cerebral Edema and Neurological Deficits in Mice After TBI

**FIGURE 7:**
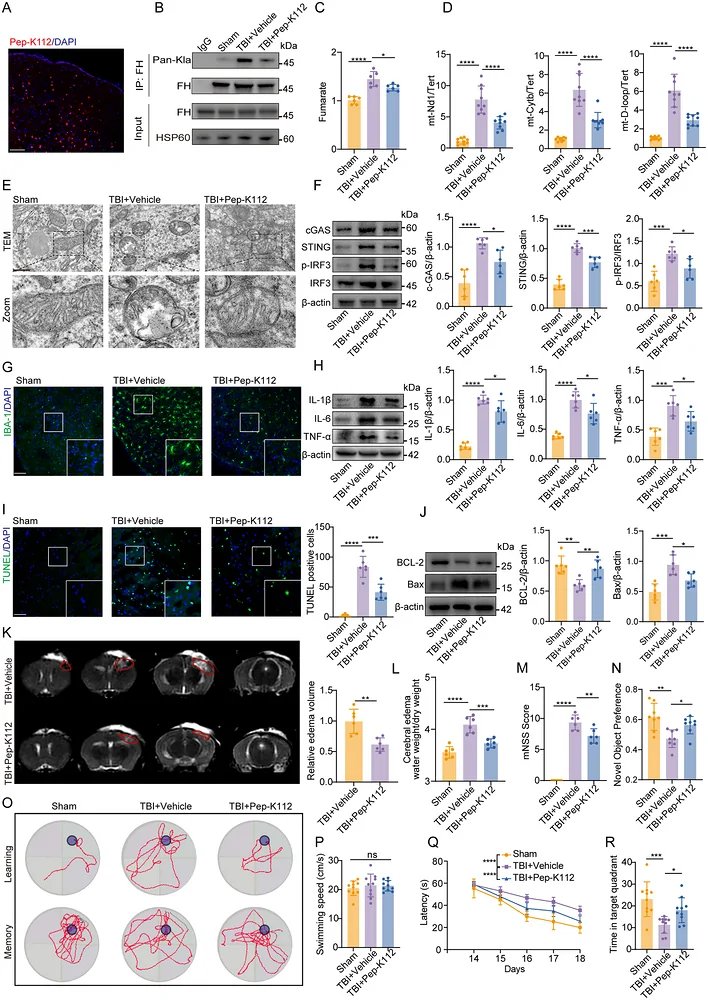
Pep‐K112 Attenuates Cerebral Edema and Neurological Deficits in Mice After TBI. (A) Representative image of Pep‐K112 in the brain of TBI mice after ICV. Scale bar, 100 µm. (B) Western blot analysis of FH lactylation levels after treatment with Pep‐K112 at 1 days after TBI. (C) Fumarate levels of mice brain tissues in indicated groups at 3 days after TBI (n = 6 per group). (D) qPCR quantification of cytosolic mtDNA (mt‐Nd1, mt‐Cytb, mt‐D‐loop) in brain tissues of each group at 3 days after TBI normalized to the nuclear DNA levels (Tert). (n = 9 per group). (E) Representative TEM and enlarged mitochondria in each group at 3 days after TBI. Scale bar, 500 nm. (F) Western blot analysis of cGAS, STING, and p‐IRF3 expression in different groups at 3 days after TBI (n = 6 per group). (G) Representative immunofluorescence images of IBA‐1 in different groups at 3 days after TBI. Scale bar, 100 µm. (H) Western blot analysis of IL‐1β, IL‐6, and TNF‐α expression in different groups at 3 days after TBI (n = 6 per group). (I) Representative images of TUNEL staining in different groups at 3 days after TBI (n = 6 per group). Scale bar, 50 µm. (J) Western blot analysis of BCL‐2 and Bax expression in different groups at 3 days after TBI (n = 6 per group). (K) MRI images and injury volume analysis in different groups at 3 days after TBI (n = 6 per group). (L) Cerebral edema assessments of mice in different groups at 3 days after TBI (n = 6 per group). (M) mNSS scores of mice in different groups at 3 days after TBI (n = 6 per group). (N) NOR of mice in different groups at 14 days after TBI (n = 8 per group). (O) Representative swimming trajectories of mice during the learning and memory phases of the MWM test. (P) Swimming speeds of mice in different groups during the MWM test after TBI (n = 10 per group). (Q) Mean latency of mice in different groups to reach the visible platform after TBI (n = 10 per group). (R) Time spent by mice in different groups in the target quadrant after TBI (n = 10 per group). Data are presented as mean ± SD. *p < 0.05, **p < 0.01, ***p < 0.001, ****p < 0.0001; ns, not significant. One‐way ANOVA followed by Bonferroni post hoc test (C‐D, F, H‐J, L‐N, P and R). Two‐tailed unpaired Student's t test (K). Two‐way ANOVA followed by Bonferroni post hoc test (Q).

To assess the in vivo therapeutic potency of Pep‐K112, we delivered the peptide via intracerebroventricular (ICV) injection. Based on cerebral edema quantification and neurological scoring, we defined the optimal therapeutic regimen: a concentration of 2 µg/µL (5 µL) administered at 6 h post‐TBI (Figure S7A–D). Immunofluorescence staining showed that, following ICV injection, Pep‐K112 was distributed across the cerebral cortex but exhibited preferential enrichment in neurons (Figure 7A and Figure S7E). Given that FH is expressed at relatively high levels in neurons and Pep‐K112 displays molecular specificity for FH, these two factors likely act in concert to drive the selective accumulation of the peptide within neuronal cells (Figure S7F,G). Consistently, Pep‐K112 administration blunted TBI‐triggered FH lactylation and fumarate accumulation and lowered cytoplasmic mtDNA abundance in damaged brain parenchyma (Figure 7B–D). Transmission electron microscopy (TEM) further demonstrated that Pep‐K112 alleviated TBI‐evoked mitochondrial swelling, cristae depletion, and membrane damage. (Figure 7E). Mechanistically, Pep‐K112 suppressed cGAS–STING cascade activation and downstream neuroinflammation, ultimately curbing neuronal apoptosis (Figure 7F–J). In line with these molecular improvements, Pep‐K112 mitigated post‐TBI cerebral edema and rescued neurological and behavioral deficits (Figure 7K–R). Notably, long‐term neurological function and brain histomorphology were also effectively restored (Figure S7H‐I).

Next, to evaluate the in vivo safety of Pep‐K112, we administered the peptide via continuous intraperitoneal (i.p.) injection (20 mg/kg) for 14 days. No significant changes in body weight were observed (Figure S7J), and no cellular damage was detected in the brain, kidney, liver, lung, or heart tissues (Figure S7K). Additionally, HE staining revealed no histological abnormalities in any of the major peripheral organs, supporting the in vivo safety profile of Pep‐K112 (Figure S7L). Collectively, these findings indicate that Pep‐K112 is safe for in vivo application and exerts neuroprotective effects by targeting FH lactylation to mitigate TBI‐induced mitochondrial damage and secondary brain injury driven by neuroinflammation (Figure S8).

## 3 Discussion

Energy metabolism disturbance and neuroinflammation constitute the core pathological events driving aggravated cerebral edema and unfavorable neurological outcomes following TBI [34, 35, 36]. In the acute phase of TBI, lactate levels increase rapidly, correlating positively with the severity of injury and poor prognosis [6, 7]. Lactate not only modulates energy metabolism but also regulates neuroinflammation through lactylation, thereby contributing to the pathological progression [10, 15]. Analysis of human and mouse snRNA‐seq datasets revealed elevated global protein lactylation after TBI. Subsequent studies in TBI mice confirmed preferential lactylation enrichment in neurons, consistent with their intrinsically high metabolic demands [37]. TBI induces ischemia and hypoxia, impairing energy production via oxidative phosphorylation [6, 7]. As an emergency energy substrate, lactate fuels neuronal energy metabolism and simultaneously generates lactyl‐CoA, which serves as a substrate for lactylation, ultimately leading to elevated lactylation levels in neurons [38, 39].

Despite the potent gene regulatory function of histones having steered research predominantly toward histone lactylation, studies have revealed that lactylation extends beyond the nucleus, exhibiting distinct subcellular compartmentalization [40, 41]. Analysis of acutely edematous brains in TBI mice revealed a significant enrichment of lactylation within mitochondria, which houses the key enzymes for the TCA cycle and oxidative phosphorylation [42, 43, 44]. Therefore, lactylation of mitochondrial proteins serves as a direct and efficient regulatory mechanism. By modifying these crucial enzymes and electron transport chain complexes, this process rapidly and dynamically alters their conformation and activity, thereby impacting ATP generation efficiency and the partitioning of metabolic intermediates [45, 46, 47]. This process establishes a direct link between lactate driven metabolic reprogramming and cellular energy homeostasis and fate. In this context, ATP and metabolic intermediates are transformed from conventional energy sources and biosynthetic precursors into key signaling molecules that drive endogenous immune responses under conditions of stress or pathology.

FH, an evolutionarily conserved mammalian enzyme, reversibly catalyzes fumarate to malate [24, 48]. This reaction is pivotal for maintaining mitochondrial oxidative phosphorylation and respiratory chain complex activity, thereby enabling ATP production [24]. Through analysis of lactylated mitochondrial proteins, we identified FH at K112 as the most significantly lactylated site after TBI. Both in vitro and in vivo experiments confirmed that lactylation at K112 leads to pathological accumulation of fumarate. This finding aligns with serum metabolomics data from TBI patients [49]. Moreover, fumarate accumulation induces mitochondrial swelling and SNX9‐dependent release of mtDNA vesicles into the cytosol, thereby activating the cGAS‐STING pathway and driving pro‐inflammatory responses [26]. This cascade disrupts the neurovascular unit, increases BBB permeability, and exacerbates vasogenic brain edema [50, 51]. FH K112 lactylation further suppresses mitochondrial ATP production and respiratory chain complex activity, disrupting mitochondrial morphology and membrane potential to induce dysfunction. This impaired ATP synthesis, particularly due to oxidative phosphorylation deficiency, compromises Na^+^‐K^+^‐ATPase activity at the plasma membrane, elevates cellular osmotic pressure, and serves as a key driver of cytotoxic edema [52]. Collectively, these factors exacerbate lactate accumulation and microenvironment acidification, fueling the progression of cerebral edema.

Here, we identified AARS2 and SIRT3 as the respective writer and eraser responsible for FH K112 lactylation. As a mitochondrial aminoacyl‐tRNA synthetase, AARS2 conventionally sustains mitochondrial homeostasis via regulating protein translation [53]. Emerging evidence has unveiled its translation‐independent function in mediating protein lactylation [47]. We further verified that AARS2 targets the mitochondrial metabolic enzyme FH, converting elevated lactate into altered FH enzymatic activity. This modification remodels TCA cycle flux and fumarate accumulation, thereby modulating downstream neuroinflammatory signaling, which provides direct evidence linking metabolic signaling to mitochondrial functional regulation. SIRT3 confers endogenous neuroprotection during TBI progression and acts as a core deacetylase and desuccinylase [31, 54, 55, 56]. Our previous study demonstrated that TBI inhibits AMPK/PGC‐1α signaling, which downregulates SIRT3 expression [57]. Consequently, TBI induced SIRT3 deficiency may be a contributor to enhanced FH lactylation. Collectively, these findings refine the regulatory mechanism of FH lactylation and offer a novel epigenetic‐metabolic insight into secondary brain injury after TBI.

Current clinical therapies for TBI remain largely symptomatic and limited, relying on decompressive craniectomy or hyperosmotic agents to reduce intracranial pressure and mitigate secondary brain injury [3, 5]. In this study, we synthesized the peptide Pep‐K112. Compared with conventional therapeutics, Pep‐K112 possesses the inherent advantages of peptide agents, including low molecular weight, excellent tissue permeability, and a favorable in vivo safety profile. Moreover, its high molecular target specificity minimizes off‐target biological effects [58]. While conferring potent neuroprotection, Pep‐K112 preserves physiological lactate metabolism and global lactylation regulatory networks, thereby enabling a wider therapeutic window and reduced adverse effects.

It is important to emphasize that, although our immunofluorescence analysis showed preferential enrichment of Pep‐K112 in neurons, this distribution pattern does not necessarily indicate cell type‐specific uptake. Cellular entry of Pep‐K112 is mediated by the TAT cell‐penetrating peptide, which generally mediates broad cellular uptake rather than receptor‐dependent targeting of a specific cell type [59]. Therefore, its preferential neuronal accumulation may reflect target‐dependent retention rather than selective uptake. This interpretation is supported by our snRNA‐seq analysis showing relatively high FH expression in neurons and by our biochemical data demonstrating molecular recognition of FH by Pep‐K112. Additional factors may also influence the neuronal distribution of Pep‐K112. TBI induces profound disturbances in cerebral energy metabolism and mitochondrial function, particularly in injured neurons [60]. Neurons also possess highly specialized cellular architecture, extensive mitochondrial networks, and substantial bioenergetic demands [61, 62]. These features, together with TBI‐associated ischemic and hypoxic stress, may alter the intracellular disposition and target engagement of Pep‐K112. Nevertheless, whether injury‐induced changes increase the accessibility of the K112‐adjacent region of FH to Pep‐K112 remains unknown. Collectively, these factors may contribute to the preferential neuronal enrichment of Pep‐K112, but the underlying mechanisms require further investigation.

This study has several limitations. First, while FH lactylation was confirmed in TBI surgical specimens, systematic omics analysis of patient brain edema samples is needed to fully elucidate its pathological impact. Second, given that FH is also expressed in the cytoplasm, the potential biological effects of cytoplasmic FH lactylation warrant further investigation. Furthermore, our study was limited to short‐ and medium‐term prognostic evaluations; future studies should involve longer‐term follow‐up to assess the long‐term efficacy and safety of Pep‐K112. Thirdly, other potential mechanisms contributing to the preferential enrichment of Pep‐K112 in neurons remain to be explored. Finally, given the limitations of ICV administration, future work will investigate on optimizing peptide structure and utilizing targeted delivery systems to enhance BBB permeability for better clinical translation.

In summary, this study reveals the regulatory role of AARS2/SIRT3‐mediated lactylation at the FH K112 site in secondary brain injury and neurological deficits following TBI, elucidating the molecular coupling mechanism between metabolic dysregulation and neuroinflammation. Furthermore, we designed Pep‐K112, a peptide specifically targeting and inhibiting FH K112 lactylation. We demonstrated that it exerts neuroprotective effects by stabilizing mitochondrial function and suppressing the hyperactivation of the cGAS‐STING pathway, thereby alleviating brain edema. These findings provide a promising molecular target and therapeutic strategy for TBI treatment via targeting metabolic and epigenetic regulation.

## 4 Methods

### 4.1 Human Brain Tissue Samples and Peripheral Blood Samples

This study was conducted in accordance with the *Declaration of Helsinki* and approved by the Tangdu Hospital Ethics Committee (No. K202410‐13). Written informed consent was obtained from all patients. Brain edematous tissues were collected from 12 patients undergoing decompressive craniectomy due to contusion or hematoma. Specific details as follows: Perilesional edematous brain tissues were collected during surgical procedures performed within 24 h after TBI onset. Upon resection, samples were washed with PBS to ensure cleanliness for subsequent processing. Control tissues were obtained from six patients undergoing craniotomy for glioma resection or cavernous hemangioma, specifically from unavoidably involved normal cortical tissues (Tables S1 and S2).

The collection and analysis of plasma from patients and volunteers was approved by the Ethics Committee of Tangdu Hospital (No. 202103‐007). After obtaining informed consent from all participants, venous blood samples were collected at admission into EDTA‐containing anticoagulant tubes. The whole blood samples were then centrifuged at 1500 × g for 10 min at 4°C to separate the plasma, which was subsequently stored at −80°C for further analysis (Table S3).

### 4.2 Animals

All C57BL/6 mice were purchased from the Experimental Animal Center of the Fourth Military Medical University. *SIRT3^flox/flox^* mice were obtained from Models Organism (Shanghai, China) and generated using CRISPR/Cas9 technology. *SIRT3^flox/flox^* mice were crossed with *Mep2^CreERT2^* mice to produce conditional *SIRT3* knockout mice specifically in *Mep2*‐positive neuronal cells. All experimental mice were male, 8–10 weeks old, weighing 25–30 g, and housed in a specific pathogen‐free environment with constant temperature and humidity, a 12‐hour light/dark cycle, and ad libitum access to food and water. The SIRT3 activator, SIRT3 activator 2 (HY‐163987, MedChemExpress), was administered at a dose of 10 mg/kg via intracerebroventricular injection 24 h prior to TBI modeling in mice [63]. The SIRT3 inhibitor, 3‐TYP (HY‐108331, MedChemExpress), was administered at a dose of 50 mg/kg via i.p. injection every other day for six consecutive days before TBI modeling in mice [64]. The therapeutic efficacy and safety of Pep‐K112 were evaluated via two administration routes: a single ICV. injection and continuous i.p. injection. The single ICV dose was 2 µg/µL with an injection volume of 5 µL, while the continuous i.p. dose was 20 mg/kg administered daily for 14 days. All animal experimental protocols were approved by the Institutional Animal Care and Use Committee of the Fourth Military Medical University (No. 20230713).

### 4.3 Controlled Cortical Impact Injury Model

Following satisfactory induction of anesthesia with 4% isoflurane, mice were immobilized in a stereotaxic frame. A craniotomy was performed at the midpoint 2.5 mm lateral to the bregma and sagittal suture. The skull cap was carefully removed to ensure the integrity of the underlying dura mater remained intact. CCI injury was induced using a controlled cortical impact device equipped with a 2 mm flat metal tip, with the following parameters: velocity of 3.0 m/s, penetration depth of 1.0 mm, and dwell time of 100 ms. Postoperatively, the incision was disinfected and sutured. Mice in the sham operation group underwent the same surgical procedures but without CCI induction.

### 4.4 Lactate Level Measurement

Lactate content in brain tissue was quantified using the L‐Lactate Content Assay Kit (S0208S, Beyotime) according to the manufacturer's protocol.

### 4.5 Immunoprecipitation and Western Blotting

#### 4.5.1 Immunoprecipitation

Protein A/G agarose beads (P2545, Sigma–Aldrich) were added to the protein solution and incubated with rotation at 4°C for 10 min. The protein concentration of the supernatant was determined using a BCA assay. Antibodies were then added at the appropriate ratio to the supernatant, and the mixture was incubated with rotation at 4°C overnight. To capture the antigen‐antibody complexes, an equal volume of fresh agarose beads was added, followed by incubation at room temperature for 2 h with gentle agitation. The beads‐bound complexes were pelleted by centrifugation and washed three times with pre‐chilled PBS. The antigen, antibody, and beads were eluted by boiling the samples in Laemmli sample buffer at 95°C for 5 min. After centrifugation, the supernatant was collected for subsequent Western blot analysis.

#### 4.5.2 Western Blot

Cells and brain tissues were lysed with RIPA lysis buffer to extract total proteins. After determining the protein concentration, equal aliquots were prepared to standardize the loading volume. Protein samples were separated by electrophoresis and then transferred onto polyvinylidene fluoride membranes. The membranes were blocked with 5% non‐fat milk for 1 h, followed by incubation with primary antibodies overnight at 4°C. Subsequently, the membranes were incubated with horseradish peroxidase (HRP)‐conjugated secondary antibodies for 1 h at room temperature. Imaging was performed using a Bio‐Rad imaging system, and quantitative analysis was conducted with ImageJ software.

#### 4.5.3 Antibodies

Pan‐Kla antibody (Rabbit, 1:1000, 1401RM, PTM Biolabs); β‐actin antibody (Rabbit, 1:5000, 20536‐1‐AP, Proteintech); HSP60 antibody (Rabbit, 1:2000, 15282‐AP, Proteintech); FH antibody (Rabbit, 1:2000, 11375‐1‐AP, Proteintech); AARS2 (Rabbit, 1:1000, 22696‐1‐AP, Proteintech); HA tag antibody (Rabbit, 1:20, 51064‐2‐AP, Proteintech); TFAM antibody (Rabbit, 1:1000, 15218, CST); cGAS antibody (Rabbit, 1:1000, 31659, CST); STING antibody (Rabbit, 1:1000, 13647, CST); p‐IRF3 antibody (Rabbit, 1:1000, 4947, CST); IRF3 antibody (Rabbit, 1:1000, ab245341, Abcam); SIRT3 antibody (Rabbit, 1:1000, 10099‐1‐AP, Proteintech); IL‐1β antibody (Rabbit, 1:1000, WL00891, Wanleibio); IL‐6 antibody (Rabbit, 1:1000, ab290735, Abcam); TNF‐α antibody (Rabbit, 1:1000, 11948, CST); BCL‐2 antibody (Rabbit, 1:1000, 26593‐1‐AP, Proteintech); Bax antibody (Rabbit, 1:2000, 50599‐2‐Ig, Proteintech).

### 4.6 Immunofluorescence Staining

Brain sections or cell coverslips were fixed with 4% paraformaldehyde, followed by incubation in blocking buffer for 1 h at room temperature. The samples were then incubated overnight at 4°C with primary antibodies as follows: Pan‐Kla Antibody (Rabbit, 1:500, 1401RM, PTM Biolabs); NeuN Antibody (Goat, 1:500, NBP3‐05554, Novus Biologicals); GFAP Antibody (Chicken, 1:500, PA1‐10004, Thermo); TOMM20 Antibody (Rat, 1:500, ab289670, Abcam); mtDNA Antibody (Mouse, 1:200, CBL186, Sigma); IBA‐1 Antibody (Rabbit, 1:200, 17198, CST). After washing, the samples were incubated with the corresponding Alexa Fluor‐conjugated secondary antibodies for 1 h at room temperature. Images were acquired using a confocal microscope (Nikon).

### 4.7 snRNA‐seq

Data acquisition and analysis of human snRNA‐seq: The snRNA‐seq data, derived from brain tissues of patients with TBI, were obtained from the NCBI Gene Expression Omnibus (GEO): GSE209552. Data acquisition and analysis of mouse snRNA‐seq:Following isolation, mouse brain tissue is gently washed in ice‐cold PBS to remove blood and debris. The tissue is then dissociated in an ice‐conical DNase‐containing enzymatic buffer, and the homogenate is sequentially filtered through a 70 µm cell strainer. The filtrate is collected by centrifugation to pellet crude nuclei. The nuclear pellet is resuspended, mixed 1:1 with 50% iodixanol solution, and gently overlaid onto a 30% iodixanol step gradient. After ultracentrifugation at 10,000 × g for 20 min at 4°C, the upper myelin layer is aspirated, and purified nuclei are harvested from the 30% iodixanol interface using a wide‐bore pipette tip. Harvested nuclei are resuspended in 10x Genomics Chromium Nuclei Buffer, counted, and adjusted to the recommended concentration before proceeding with downstream workflows (nuclei capture, reverse transcription, cDNA amplification, and library preparation) per the manufacturer's standardized protocols. Raw reads were preprocessed using Cell Ranger (version 5.0.0, 10× Genomics)) with the default parameters and aligned to the pre‐mRNA reference (Ensemble_release 105, Mus musculus). For quality control, cells with gene counts > 200 per cell, UMI counts < 8000 per cell, and a percentage of mitochondrial genes < 25% were retained for downstream analysis.

The data were integrated using the Harmony algorithm (RunHarmony, Seurat, Version 4.1.0), and integrated UMAP dimensionality reduction results were generated. During the visualization and clustering analysis phase, a manifold was constructed via the RunUMAP function of the Seurat package. Meanwhile, cell clustering was accomplished using the shared nearest neighbor algorithm (FindClusters, Seurat) combined with a module optimization strategy. Annotation of the obtained cell clusters was implemented according to the expression characteristics of canonical marker genes. Additionally, the AddModuleScore function of the Seurat package was employed to calculate the enrichment scores for the genes related to selected lactylation‐related pathways.

### 4.8 Lysine Lactylome Analysis

To identify endogenous lactylated proteins and accurate modification sites after TBI, the collected brain edema tissues were ground and mixed with lysis buffer. After ultrasonic lysis, the supernatant was collected to determine the protein concentration. Equal amounts of proteins from the sham group and TBI group were subjected to proteolysis, followed by precipitation with trichloroacetic acid. The precipitate was washed with acetone, dried, and then incubated with trypsin for overnight enzymatic hydrolysis. After reduction with dithiothreitol, iodoacetamide was added, and the mixture was incubated at room temperature in the dark. The peptides were dissolved in immunoprecipitation buffer, and the supernatant was transferred to lactylation resin (PTM‐1404; PTM‐Bio) for overnight incubation. The peptides bound to the resin were eluted for LC‐MS/MS analysis.

### 4.9 Lactylation Score

Based on snRNA‐seq data, we utilized the AddModuleScore function embedded in Seurat (v 5.3.1) to quantify the activity of protein lactylation‐related genes in each nucleus. Genes closely associated with lactylation were screened from the MSigDB public database to construct a lactylation feature gene set (Table S5). This set includes genes involved in lactate metabolism and transport, lactylation regulation, and lactylated target protein coding.

The AddModuleScore function was run with default parameters (nbin = 24), selecting 100 control genes randomly from each expression bin to correct for background expression levels. The final lactylation score was calculated as the difference between the gene set score and the background score. This score serves to reflect the co‐expression level of lactylation‐related genes and acts as a surrogate metric for the potential activity of lactylation modification.

### 4.10 Mitotracker Staining

To assess mitochondrial morphology, cells were stained using the Mitochondrial Green Fluorescent Staining Kit (C‐1048, Beyotime) according to the manufacturer's protocol. Mitochondrial morphological changes were subsequently observed and imaged under a confocal microscope.

### 4.11 Immunohistochemical Staining

Brain tissues were fixed in 4% paraformaldehyde and then cut into 5 µm frozen sections. After fixation with pre‐chilled acetone, endogenous peroxidase was inactivated with 3% hydrogen peroxide. Following blocking, the sections were incubated overnight at 4°C with the primary antibody: Rabbit anti‐FH Antibody (1:250, 11375‐1‐AP, Proteintech). After washing, the sections were incubated with Biotin‐conjugated Goat Anti‐Rabbit IgG (1:200, SA00004‐2, Proteintech) for 20 min at 37°C. Images were acquired using a confocal microscope.

### 4.12 JC‐1 Staining

After cell treatment under different intervention conditions, mitochondrial membrane potential was detected using the Enhanced Mitochondrial Membrane Potential Assay Kit with JC‐1 (C2003s, Beyotime). The assay was performed according to the manufacturer's protocol. Images were acquired under a confocal microscope to evaluate changes in mitochondrial membrane potential.

### 4.13 Deep‐kla

The prediction and analysis of lysine lactylation sites were performed based on the Deep‐Kla platform, with the web‐based online server available at: http://lin‐group.cn/server/DeepKla/.22

### 4.14 Cell Culture and Treatment

HT22 cells were obtained from Procell System (Wuhan, China) and authenticated via STR profiling. Plasmids and siRNAs were purchased from Hanheng Biotechnology (Shanghai, China). Cells were cultured in high‐glucose DMEM supplemented with 10% FBS at 37°C in a humidified incubator with 5% CO_2_. Cells grown to ∼80% confluency were transfected with corresponding plasmids and siRNA using Lipofectamine 3000 (L3000015, Invitrogen) according to the manufacturer's protocol. For drug treatments, cells were incubated with: 100 mM sodium lactate (A604046, Sangon Biotech); 1 µM AR‐C155858 (HY‐13248, MedChem Express); 9 µM FX11 (HY‐16214, MedChem Express); 10 µM CBP inhibitor (SGC‐CBP30, S7256, Selleckchem), 5 µM GLO1 inhibitor (BrBzGCP2, HY‐136684, MedChemExpress), 5 µM SNX9 inhibitor (DATPT, HY‐145307, MedChemExpress), 20 µM Pep‐K112 for 24 h. The SIRT3 activator 5‐Heptadecylresorcinol (HY‐N2673, MedChem Express) and SIRT3 inhibitor 3‐TYP (HY‐108331, MedChem Express) were administered at 5 µM for 4 h prior to lactate intervention [65].

### 4.15 The Peptide Sequences

Peptide‐1 (Pep‐K112): RKKRRQRRR‐GGG‐EYGLDPKIASAIM‐GGG‐TAMRA; Peptide‐2: RKKRRQRRR‐GGG‐NQEYGLDPKIASA‐GGG‐TAMRA; Peptide‐3: RKKRRQRRR‐GGG‐GLDPKIASAIMKA‐GGG‐TAMRA; Tag Control: RKKRRQRRR‐GGG‐YPYDVPDYA; K112 mutant Control: RKKRRQRRR‐GGG‐ EYGLDPRIASAIM‐GGG‐YPYDVPDYA; Peptide‐K112 with Tag: RKKRRQRRR‐GGG‐EYGLDPKIASAIM‐GGG‐YPYDVPDYA.

### 4.16 Immunoprecipitation Coupled With Mass Spectrometry

HT22 cells were lysed in NP‐40 lysis buffer supplemented with a protease inhibitor cocktail. Cell lysates were centrifuged at 20,000 × g for 20 min at 4°C, and the resulting supernatant was incubated with anti‐FH antibody overnight at 4°C with gentle rotation. Subsequently, Protein A/G magnetic beads were added to the mixture and incubated for 3 h to capture antibody‐protein complexes. The beads were thoroughly washed three times with lysis buffer containing Triton X‐100 to remove non‐specifically bound proteins. Bound proteins were eluted from the beads, followed by in‐solution trypsin digestion. The digested peptides were acidified with 0.15% trifluoroacetic acid (TFA) and prepared for subsequent LC‐MS/MS analysis.

To verify the targeting specificity of Pep‐K112, we performed a peptide pull‐down assay coupled with LC‐MS/MS analysis. Cell lysates were prepared in ice‐cold lysis buffer supplemented with protease inhibitors. Equal amounts of protein were incubated with Pep‐K112 and two control peptides (tag control and K112 mutant control) overnight at 4°C. Peptide‐protein complexes were captured using magnetic beads and thoroughly washed to remove non‐specific contaminants. The captured proteins were eluted, digested with trypsin, and subjected to LC‐MS/MS analysis. The enrichment of HA peptide in each pull‐down group was assessed to evaluate the targeting specificity of Pep‐K112.

### 4.17 Fumaric Acid Level Measurement

Intracellular and tissue fumarate levels were quantified using the Fumarate Assay Kit (YJ100028, Mlbio). Cell samples were rinsed with PBS and lysed in ice‐cold lysis buffer for 30 min. Tissue specimens were weighed, homogenized in lysis buffer (1:10 w/v) on ice, and centrifuged (12,000 rpm, 20 min, 4°C). The supernatant was collected for analysis, with all subsequent steps executed per the manufacturer's protocol. Fumarate levels are expressed as fold change relative to the WT or sham group.

### 4.18 ATP Level Measurement

Cells were cultured under various conditions for 24 h and lysed with lysis buffer on ice for 30 min. Subsequently, samples were centrifuged at 12,000 rpm for 10 min at 4°C. The supernatant was collected for ATP measurement using the ATP Assay Kit (S0026, Beyotime). ATP detection was performed strictly according to the manufacturer's instructions.

### 4.19 Mitochondrial Isolation and Respiratory Chain Complex Activity Assay

Mitochondrial isolation and protein extraction were performed using a Mitochondrial Isolation and Protein Extraction Kit (PK10016, Proteintech) strictly in accordance with the manufacturer's instructions. The activities of mitochondrial respiratory chain Complexes I, II, III, IV, and V were determined using the corresponding commercial assay kits (E‐BC‐K834‐M, E‐BC‐K835‐M, E‐BC‐K836‐M, E‐BC‐K837‐M, E‐BC‐K838‐M; Elabscience), following the manufacturer's protocols for each kit.

### 4.20 Oxygen Consumption Rate (OCR) Measurements

HT22 cells were seeded into Seahorse XF 96‐well cell culture plates at a density of 1×10^4^ cells per well and cultured overnight in a 37°C, 5% CO_2_ incubator to allow for complete adhesion. Subsequently, the cells were divided into different groups and subjected to the corresponding treatments. After 24 h of intervention, the culture medium was replaced with pre‐warmed Seahorse XF Base Medium, and the cells were incubated in a CO_2_ free incubator at 37°C for 1 h to equilibrate.

OCR were measured using a Seahorse XF96 Extracellular Flux Analyzer according to the manufacturer's instructions. The sequential injection of mitochondrial inhibitors was performed as follows: 1 µM oligomycin, 1 µM carbonyl cyanide 4‐(trifluoromethoxy) phenylhydrazone (FCCP), and 1 µM rotenone plus 1 µM antimycin A. Following the assay, the total protein concentration of cells in each well was determined using a BCA protein assay kit, and the OCR values were normalized to the protein concentration to eliminate variations caused by differences in cell number.

### 4.21 Transmission Electron Microscopy

For cell samples, the medium was discarded from cells treated under different conditions. After fixation with 2.5% glutaraldehyde at room temperature, the cells were gently harvested into centrifuge tubes and centrifuged at 2,500 rpm; the resulting cell pellets were retained. For cerebral cortical tissue samples, immediately after ex vivo isolation, the tissues were dissected into 1 mm^3^ blocks and fixed with glutaraldehyde overnight. Subsequently, all samples were subjected to post‐fixation with 1% osmium tetroxide, dehydration through a graded ethanol series, and acetone immersion, followed by resin embedding. After embedding, ultrathin sections (50–70 nm) were cut, stained with uranyl acetate and lead citrate, and finally imaged using a transmission electron microscope (Hitachi).

### 4.22 Quantitative Proteomics Analysis

HT22 cells were divided into two groups: Lactate and Lactate + Pep‐K112. After corresponding treatments, cells were collected and lysed in ice‐cold lysis buffer containing protease inhibitor cocktail. Total protein was extracted and quantified, and equal amounts of protein from each group were subjected to in‐solution trypsin digestion. The resulting peptide mixtures were desalted, lyophilized, and reconstituted for LC‐MS/MS analysis.

Raw mass spectrometry data were acquired on a high‐resolution mass spectrometer and searched against the mouse protein database using professional proteomics software. Protein identification and quantitative analysis were performed with a false discovery rate (FDR) < 0.01 at both peptide and protein levels.

Functional gene sets for two core biological pathways (TCA cycle and acute inflammatory response) were constructed by retrieving corresponding standard GO terms from the AmiGo 2 database, obtaining GO IDs, and extracting manually curated Mus musculus protein lists from the UniProt Knowledgebase. After matching these gene sets with the quantitative proteomic data to extract identified proteins, Z‐score normalization and hierarchical clustering were performed using OmicStudio tools to generate global heatmaps for visualizing pathway‐specific protein expression patterns across groups.

### 4.23 Real‐Time Quantitative PCR

Total RNA was extracted using TRIzol reagent (279510, Thermo Fisher Scientific). After quantification with a NanoDrop 2000C spectrophotometer (Thermo Fisher Scientific), reverse transcription was performed using the Transcriptor First Strand cDNA Synthesis Kit (Roche). qPCR was carried out with SYBR Green Premix (Roche) as the fluorescent dye. The relative gene expression level was normalized with the β‐actin using the comparative cycle threshold (Ct) method (2^−ΔΔCt^). Primer sequences used for this study: *Slc25a4*: 5′‐AGAGTGTGACAGCCGTTGC‐3′, 5′‐ACCTTTGAAGAAAGCGTTGGC‐3′. *Fh*: 5′‐GCAGTGGAAGTTCACAAGGTCCTG‐3′, 5′‐CTGGACTTGCTGAACGTAACCAC‐3′. *Atp5f1a*: 5′‐GCCCTCGGTAATGCTATTGA‐3′, 5′‐GCAATCGATGTTTTCCCAGT‐3′. *Sdha*: 5′‐TTTCAGAGACGGCCATGATCT‐3′, 5′‐TGGGAATCCCACCCATGTT‐3′. *Atp6v1b2*: 5′‐GGCCCACAGAGAATCAGGTA‐3′, 5′‐GAGGGTGGGATGTAGGGTTT‐3′. *Mdh2*: 5′‐TTCAACACCAACGCTACCATTGTG‐3′, 5′‐GTGTTCGCTCTGACGATGTCAAGG‐3′. *Sucla2*: 5′‐ACCCTTTCGCTGCATGAATAC‐3′, 5′‐CCTGTGCCTTTATCACAACATCC‐3′. *Aco2*: 5′‐TGATGCAAACCCTGAGACC‐3′, 5′‐GAGCCTCCAACTTGAACTTCT‐3′. *β‐Actb*: 5′‐GTGACGTTGACATCCGTAAAGA‐3′, 5′‐GCCGGACTCATCGTACTCC‐3′.

To analyze mtDNA copy number in the cytoplasm, cells were divided into two equal portions: one portion for cytoplasmic isolation using a protocol established in previous studies [66, 67], followed by quantification of cytoplasmic mtDNA copies, and the other portion for total cellular DNA extraction via the DNA Extraction Kit (DP304, TIANGEN) as per manufacturer's instructions. Subsequently, the cytoplasmic‐to‐total mtDNA copy number ratio was calculated.″ Primer sequences used for this study:*D‐loop*: 5′‐AATCTACCATCCTCCGTGAAACC‐3′, 5′‐TCAGTTTAGCTACCCCCAAGTTTAA‐3′. *mt‐Cytb*: 5′‐GCTTTCCACTTCATCTTACCATTTA‐3′, 5′TGTTGGGTTGTTTGATCCTG‐3′. *mt‐ND1*:5′‐CTAGCAGAAACAAACCGGGC‐3′, 5′‐CCGGCTGCGTATTCTACGTT‐3′. *Tert*: 5′‐CTAGCTCATGTGTCAAGACCCTCTT‐3′, 5′‐GCCAGCACGTTTCTCTCGTT‐3′.

### 4.24 Cell Viability

Cell Counting Kit‐8 (C0037, Beyotime) was employed to assess cell viability. For the CCK‐8 assay, cells were treated with Pep‐1, Pep‐2, and Pep‐3 at a concentration of 20 µM for 24 h, and the experimental procedure was carried out in strict accordance with the manufacturer's instructions.

### 4.25 HE Staining

Freshly isolated brain tissues were fixed in 4% paraformaldehyde solution for 24 h at 4°C to preserve tissue architecture and cellular morphology. Following thorough rinsing with PBS, the fixed tissues were dehydrated through a graded ethanol series, cleared in xylene, and embedded in molten paraffin wax. The paraffin‐embedded tissue blocks were sectioned into 6 µm thick slices using a microtome; these sections were then subjected to standard deparaffinization and rehydration procedures before being stained with hematoxylin and eosin for histological observation.

### 4.26 Nissl Staining

Brain sections were deparaffinized, rehydrated through graded ethanol, and rinsed in distilled water. Sections were stained with 1% toluidine blue O at room temperature for 15 min, rinsed briefly, and differentiated in 70% ethanol under microscopic supervision until Nissl bodies appeared deep blue with a clear background. After dehydration in graded ethanol, clearing in xylene, and mounting with neutral balsam, neuronal morphology and survival were observed under light microscopy.

### 4.27 Molecular Docking

AlphaFold‐predicted structures for FH (ID: P97807) and SIRT3 (ID: Q8R104) were downloaded from the UniProt database (https://www.uniprot.org/). The HawkDOCK SERVER (http://cadd.zju.edu.cn/hawkdock/) was utilized for protein‐protein molecular docking and binding free energy calculation. Docking Score and Binding free energy served as evaluation parameters, with results visualized using PyMOL software.

To evaluate the changes in binding free energy of the FH protein before and after lactylation at the K112 site, PyMOL software (version 3.1.3), Gaussian software (version 16), and GaussView software (version 6.0) were used to introduce the lactylation modification at this residue. During this process, Sobtop software (version 1.0(dev5)) was employed to generate the topology file for the non‐standard amino acid residue. The structural model of the modified FH protein was subjected to molecular dynamics simulation using GROMACS software (version 2024.3). ClusPro software (version 2.0) was utilized to perform molecular docking between the FH protein (both with and without lactylation modification) and the Sirt3 protein, resulting in the formation of protein‐protein complexes. After further MD simulation of these complexes, MMPBSA.py (version 16.0) was used to calculate the binding free energy of the protein‐protein complexes.

Molecular docking between fumaric acid (PubChem CID: 444972) and FH protein was performed using AutoDock Vina 1.1.2. The chemical structure of fumaric acid was downloaded from the PubChem database (https://pubchem.ncbi.nlm.nih.gov/). ChemDraw 20.0 was applied to construct the three‐dimensional structure of the compound and conduct energy minimization. AutoDock Tools 1.5.6 was used to generate PDBQT files for subsequent docking simulation. The docking conformation with the lowest binding energy and highest cluster population was selected as the optimal binding mode between ligand and protein. Docking results were expressed as binding energy values, with experiments performed in triplicate to calculate the mean value and standard deviation. Finally, PLIP (https://plip‐tool.biotec.tu‐dresden.de/) and PyMOL 2.4 were adopted for visualization of docking outcomes.

### 4.28 TUNEL Staining

TUNEL assays were conducted on brain sections using the commercial TUNEL Apoptosis Detection Kit (E‐CK‐A320, Elabscience) as per the manufacturer's instructions. After chemical fixation, samples were visualized via confocal microscopy, and apoptotic nuclei (TUNEL‐positive) were quantified using ImageJ software.

### 4.29 MRI

Brain tissue injury in mice was assessed using a 3T MRI scanner. T2‐weighted imaging was employed to evaluate total lesion volume. The lesion volume was calculated using the formula: V = ∑ [(anteroposterior diameter × transverse width) × slice thickness]. Data were analyzed by two investigators in a blinded manner.

### 4.30 Cerebral Edema Measurement

Three days post‐TBI modeling in mice, animals were euthanized via decapitation under anesthesia. Brain tissues were harvested, and the ipsilateral and contralateral cerebral hemispheres were isolated. Each hemisphere was placed on pre‐weighed slides, weighed, and recorded. Samples were then dried in an oven at 80°C for 72 h. Tissue dry weight was measured, and brain edema was assessed using the wet‐to‐dry weight ratio.

### 4.31 Modified Neurological Severity Score

The modified neurological severity score (mNSS) was used to evaluate the degree of neurological deficit in mice. This scoring system incorporates a comprehensive neurological assessment covering multiple domains, including motor function, sensory function, and reflex function, with a total score of 18 points. A higher score indicates a more severe neurological deficit.

### 4.32 Morris Water Maze Test

The Morris water maze test was performed to evaluate spatial memory in mice. The apparatus consisted of a circular pool with a diameter of 120 cm and a height of 50 cm, along with a hidden escape platform (10 cm in diameter) submerged 1 cm below the water surface; the water temperature was maintained at 23–24°C throughout the experiment. Prior to the formal training phase, a two‐day acclimatization period was implemented, during which mice were allowed to swim freely for 60 s per session to habituate to the testing environment. In the acquisition phase, mice were trained to locate the hidden escape platform over five consecutive days, with four trials conducted daily and each trial lasting 60 s. Afterwards, the hidden platform was removed for the probe trial; the total duration of 60 s was recorded, and the time that mice spent in the target quadrant was quantified for spatial memory assessment.

### 4.33 Novel Object Recognition (NOR) Test

The NOR test was performed to evaluate cognitive memory. Mice were habituated to the open field for 10 min daily for 2 consecutive days. On day 3, the training phase involved placing two identical objects symmetrically in the arena, allowing mice to explore freely for 5 min. 24 h later, the test phase was conducted by replacing one familiar object with a novel one, and exploratory behavior was recorded for 5 min. All sessions were conducted under dim light, and the apparatus and objects were cleaned with 75% ethanol between trials to eliminate olfactory cues. Exploration was defined as sniffing or touching the object with the nose or forepaws. Recognition Index = T*novel /* (T*novel* +T*familiar*) ×100%.

### 4.34 Statistical Analysis

All data were expressed as mean ± standard deviation (SD). Comparisons between two groups were performed using unpaired Student's *t*‐tests. Pearson correlation analysis was used to assess the relationship between two variables, with correlation coefficients *r* and *p*‐values indicated in the figures. For univariate comparisons among multiple groups, one‐way analysis of variance (ANOVA) followed by the Bonferroni post‐hoc test was applied. For comparisons involving two or more variables across multiple groups, two‐way ANOVA followed by the Bonferroni post‐hoc test was used. Statistical analyses were conducted using GraphPad Prism 9.0 software. Statistical significance was defined as *p* < 0.05.

## Author Contributions

Y.Q., J.Z., and D.F. conceived and directed the study. Y.T., S.M., and B.Y. designed and performed most of the experiments. F.G., H.G., H.L., X.W., and W.C. were responsible for data acquisition and analysis. J.D., Q.L., and Y.G. conducted mice experiments and genotyping. J.M., Y.Z., and J.Z. (Jiazhen Zhao) performed the bioinformatic analysis of LC/MS and snRNA‐seq data. D.X., J.T. and Z.Z. were responsible for the cell cultures. K.W. and M.X. clinical data analysis immunofluorescence staining on human brain samples. Y.T., B.Y. and S.M. prepared the manuscript and figures.

## Conflicts of Interest

The authors declare no conflicts of interest.

## Supporting information




**Supporting File 1**: advs77698‐sup‐0001‐SuppMat.docx.

**Supporting File 2**: advs77698‐sup‐0002‐S4.xlsx.

**Supporting File 3**: advs77698‐sup‐0003‐S5.xlsx.

## Data Availability

The data that support the findings of this study are available from the corresponding author upon reasonable request.
