# Supplementary material for: Fumarate Hydratase Lactylation Exacerbates Traumatic Brain Injury Pathology via Mitochondrial Dysfunction and Neuroinflammation
Source: Adv Sci (Weinh). 2026 Sep 27:e77698. Online ahead of print. doi: 10.1002/advs.77698 (PMC13616241; doi:10.1002/advs.77698)
Supplement: Supplementary file 1 [file ADVS-9999-e77698-s003.docx]

**Supplementary Materials**

**Figure S1. Lactylation levels elevated following TBI**

(A) Dot plots show the expression of typical marker genes in various cell types from control and TBI patients.

(B) Global lactylation scores of different cell types from snRNA-seq data in control and TBI patients.

(C) Dot plots show the expression of typical marker genes in various cell types from sham and TBI mice.

(D) Lactylation scores of different cell types from snRNA-seq data in sham and TBI mice.

(E) Representative immunofluorescence images showing co-localization of Pan-Kla with neurons, microglia and astrocytes in brain tissue from sham and TBI mice. Scale bar, 100 μm.

**P* < 0.05, ***P* < 0.01, ****P* < 0.001, *****P* < 0.0001. Two-sided unpaired Wilcoxon test (B, D).

**Figure S2. Mitochondrial protein lactylation is elevated after TBI**

(A) Western blot and quantification of mitochondrial protein lactylation levels in sham and TBI mice (n = 6 per group).

(B) Western blot and quantification of mitochondrial protein lactylation levels in control and TBI patients (n = 4 per group).

(C) Western blot and quantification of mitochondrial protein lactylation levels of HT22 cells in different groups (AR-C155858, 1μM, FX11, 9μM, n = 3 independent experiments).

(D) Representative staining images and analysis of mitochondria in different groups. Scale bar, 5 μm. (n = 30 individual mitochondria).

(E) Reactome enrichment analysis of differentially expressed mitochondrial lactylated proteins in sham and TBI mice.

(F) KEGG enrichment analysis of differentially expressed mitochondrial lactylated proteins in sham and TBI mice.

(G) WikiPathways enrichment analysis of differentially expressed mitochondrial lactylated proteins in sham and TBI mice.

Data are presented as mean ± SD. **P* < 0.05, ***P* < 0.01, *****P* < 0.0001. Two-tailed unpaired Student’s t test (A-B). One-way ANOVA followed by Bonferroni post hoc test (C-D).

**Figure S3. FH is lactylated at K112 after TBI**

(A) Relative quantification of target protein expression in mitochondria via MS/MS.

(B) qPCR quantification of target gene expression (n = 3 independent experiments).

(C) Representative immunohistochemical staining of FH in brain tissue from control and TBI patients. Scale bar, 100 μm.

(D) Potential lactylation sites of FH predicted by Deep-Kla.

(E) Schematic diagram of FH crystal structure and lysine 112 residue.

(F) Lactate content in brain tissue of mice at indicated time points after TBI (n = 6 per group).

(G) Western blot analysis of FH lactylation levels in brain tissue of mice at indicated time points after TBI (n = 6 per group).

Data are presented as mean ± SD. **P* < 0.05, ***P* < 0.01, ****P* < 0.001, *****P* < 0.0001, ns, not significant. Two-tailed unpaired Student’s t test (A-B). One-way ANOVA followed by Bonferroni post hoc test (F-G).

**Figure S4. FH K112 lactylation impairs mitochondrial function and triggers mtDNA release**

(A) Molecular docking simulation and binding energy between FH and fumaric acid.

(B) Activity of mitochondrial respiratory chain complexes I, II, III, IV and V in HT22 cells in indicated groups (n = 3 independent experiments).

(C) Representative staining images and analysis of mitochondria in indicated groups. Scale bar, 5 μm. (n = 30 individual mitochondria).

(D) Representative ultrastructures of mitochondrial details in each group. Scale bar, 500 nm.

(E) qPCR quantification of cytosolic mtDNA (mt-Nd1, mt-Cytb, mt-D-loop) in HT22 cells, normalized to nuclear DNA (Tert) from whole-cell lysates (n = 3 independent experiments).

(F) Western blot analysis of TFAM expression in the cytosol and mitochondria in indicated groups (SNX inhibitor 5μM, n = 3 independent experiments).

Data are presented as mean ± SD. **P* < 0.05, ***P* < 0.01, ****P* < 0.001, *****P* < 0.0001. One-way ANOVA followed by Bonferroni post hoc test (B-C and E-F).

**Figure S5. AARS2 and SIRT3 regulate FH K112 lactylation as “writer” and “eraser”**

(A) Western blot analysis of FH lactylation levels in HT22 cells following knockdown of AARS2.

(B) Co-IP analysis of AARS2 in mitochondrial lysates of mouse brain tissue.

(C) Western blot analysis of FH lactylation levels in HT22 cells following with CBP inhibitor (10μM).

(D) Western blot analysis of FH lactylation levels in HT22 cells following with GLO1 inhibitor (5μM).

(E) Western blot analysis of Pan-Kla levels in each group of HT22 cells following overexpression of SIRT3, SIRT4 or SIRT5.

(F) Western blot analysis of Pan-Kla levels in each group of HT22 cells (SIRT3 inhibitor, 5μM).

(G) Co-IP analysis of SIRT3 in mitochondrial lysates of mouse brain tissue.

(H) Western blot analysis of FH lactylation levels in indicated groups of HT22 cells (SIRT3 inhibitor, 5μM).

(I) Western blot analysis of FH lactylation levels in indicated groups of HT22 cells (SIRT3 inhibitor, 5μM).

(J) Western blot analysis of FH lactylation levels in indicated groups of mice after TBI.

(K) Western blot analysis of FH lactylation levels in indicated groups of mice after TBI.

(L) Molecular docking simulation and binding energy between FH and SIRT3.

(M) Western blot analysis of SIRT3 expression in sham and TBI mice (n = 6 per group).

(N) Genotyping of MAP2-SIRT3 conditional knockout mice.

(O) qPCR measurement of cytosolic mtDNA (mt-Cytb, mt-D-loop) in different groups of mice (n = 6 per group).

Data are presented as mean ± SD. ***P* < 0.01, *** *P* < 0.001, **** *P* < 0.0001. ns, not significant. Two-tailed unpaired Student’s t test (M). One-way ANOVA followed by Bonferroni post hoc test (O).

**Figure S6. Pep-K112 inhibits the lactylation of FH K112**

(A) Representative immunofluorescence images of mitochondria (TOMM20) and Pep1/2/3 (TRAMA) in HT22 cells. Scale bar, 50 μm.

(B) CCK-8 assay for cell viability of Pep-1/2/3 in HT22 cells. (n = 3 independent experiments).

(C) FH enzymatic activity assay with varying concentrations of Pep-K112 (n = 6 independent experiments).

(D) Fumarate levels of HT22 cells with varying concentrations of Pep-K112 (n = 6 independent experiments).

(E) Activity of mitochondrial respiratory chain complexes I, II, III, IV and V in HT22 cells of indicated groups (n = 3 independent experiments).

(F) Representative staining images and analysis of mitochondria in indicated groups. Scale bar, 5 μm. (n = 30 individual mitochondria).

(G) Representative ultrastructures of mitochondrial in indicated group. Scale bar, 500 nm.

(H) Schematic flowchart for peptide specificity analysis.

(I) Western blot detection of HA-tagged enriched on FH by indicated peptides.

(J) Western blot analysis of HA-tagged enriched on FH after HA-Pep-K112 treatment in cells transfected with wild-type or K112 mutant FH plasmids.

(K) Volcano plot displaying the proteomic profile of the HA-Pep-K112 pulldown assay. (The X-axis shows the Log_2_ fold change of protein abundance between the Pep-K112 group and the HA-tag control group. The Y-axis represents the −Log_10_ transformed signal intensity. Proteins located on the far right (positive fold change) are specifically enriched by Pep-K112, while those near the center represent non-specific background or HA-tag binders excluded by the control).

(L) GSEA results showed that the TCA cycle was activated and negative regulation of inflammatory response was enhanced in HT22 cells treated with Pep-K112.

(M) Heatmap analysis showed increased levels of TCA cycle-related proteins and reduced expression of acute inflammatory response proteins in HT22 cells treated with Pep-K112.

Data are presented as mean ± SD. * *P* < 0.05, ** *P* < 0.01, *** *P* < 0.001, **** *P* < 0.0001. ns, not significant. One-way ANOVA followed by Bonferroni post hoc test (B and D-F).

**Figure S7. Efficacy and safety evaluation of Pep-K112**

(A) Cerebral edema in TBI mice 3 days after treatment with different doses of Pep-K112 (n = 6 per group).

(B) mNSS scores in TBI mice 3 days after treatment with different doses of Pep-K112 (n = 6 per group).

(C) Cerebral edema in TBI mice 3 days after Pep-K112 treatment at different time points (n = 6 per group).

(D) mNSS scores in TBI mice 3 days after Pep-K112 treatment at different time points (n = 6 per group).

(E) Representative immunofluorescence images showing co-localization of Pep-K112(TRAMA) with neurons, microglia and astrocytes in mice brain tissue. Scale bar, 50 μm.

(F) FH expression across cell types in human snRNA-seq data.

(G) FH expression across cell types in mouse snRNA-seq data.

(H) Long-term morphological assessment in TBI mice after Pep-K112 treatment (n = 6 per group), Scale bar, 100 μm.

(I) Long-term mNSS scores in TBI mice after Pep-K112 treatment (n = 6 per group).

(J) Body weight of mice injected intraperitoneally with Pep-K112 at 20 mg/kg daily for 14 consecutive days (n = 6 per group).

(K) Assays of ALB, ALP, ALT, AST, BUN, CRE, UA, TBIL, TP, TBA, BNP, and cTNT levels in the plasma of mice treated with Pep-K112 at 20 mg/kg daily for 14 consecutive days (n = 6 per group). ALB, albumin; ALP, alkaline phosphatase; ALT, alanine aminotransferase; AST, aspartate aminotransferase; BUN, blood urea nitrogen; CRE, creatinine; UA, uric acid; TBIL, total bilirubin; TP, total protein; TBA, total bile acid; BNP, brain natriuretic peptide; cTNT, cardiac troponin T.

(L) HE staining of the brain, kidney, liver, lung, and heart in mice treated with Pep-K112 at 20 mg/kg daily for 14 consecutive days. Scale bar, 50 μm.

Data are presented as mean ± SD. * *P* < 0.05, ** *P* < 0.01, *** *P* < 0.001, **** *P* < 0.0001. ns, not significant. One-way ANOVA followed by Bonferroni post hoc test (A-D). Two-way ANOVA followed by Bonferroni post hoc test (I-J). Two-tailed unpaired Student’s t test (H and K).

**Figure S8. Schematic diagram of this study**

**Table S1. Basic characteristics of TBI patients**

| Patient Number | Sex | Age | Surgery | Admission GCS Score | mRS Score at 6 Months After Discharge | Lactate Content (mmol/L) | Pan-kla Positive Cells/FOV |
| --- | --- | --- | --- | --- | --- | --- | --- |
| 1 | male | 74 | No | 12 | 0 | 0.9023575 | - |
| 2 | male | 37 | No | 13 | 0 | 0.65061 | - |
| 3 | male | 50 | yes | 5 | 5 | 2.29115 | 26 |
| 4 | male | 55 | No | 14 | 0 | 0.9655925 | - |
| 5 | female | 74 | No | 15 | 0 | 0.27597 | - |
| 6 | male | 74 | yes | 5 | 6 | 3.24326 | 31 |
| 7 | male | 38 | yes | 3 | 6 | 3.9042475 | 22 |
| 8 | male | 59 | No | 14 | 0 | 1.922475 | - |
| 9 | male | 51 | yes | 7 | 2 | 4.8694825 | 18 |
| 10 | female | 74 | No | 13 | 1 | 1.8413425 | - |
| 11 | male | 49 | yes | 11 | 1 | 0.4048275 | 11 |
| 12 | female | 61 | No | 12 | 2 | 1.4774425 | - |
| 13 | male | 65 | yes | 11 | 0 | 0.8952 | 18 |
| 14 | male | 60 | yes | 12 | 0 | 2.5691475 | 12 |
| 15 | male | 20 | yes | 5 | 3 | 2.63119 | 26 |
| 16 | male | 52 | yes | 6 | 2 | 2.973615 | 39 |
| 17 | male | 59 | yes | 5 | 5 | 4.9076625 | 21 |
| 18 | male | 64 | yes | 12 | 1 | 2.950945 | 15 |
| 19 | male | 15 | No | 13 | 1 | 1.9439525 | - |
| 20 | male | 60 | yes | 8 | 3 | 3.4735325 | 28 |

Brain edematous tissues were collected from 12 TBI patients who required surgical intervention due to cerebral contusion or intracranial hematoma. Perilesional edematous brain tissue was collected intraoperatively. The tissue was carefully dissected and washed with PBS to remove surface debris and visible contaminants. After this cleaning process, the specimens were used for subsequent experiments. The inclusion criteria for TBI patients were: 1) age > 18 years; 2) no preoperative treatment prior to surgery; 3) surgery performed within 24 hours of injury.

**Table S2. Basic characteristics of control patients**

| Control Patients | Sex | Age | Diagnosis | |
| --- | --- | --- | --- | --- |
| 1 | female | 58 | | Oligodendroglioma |
| 2 | male | 56 | | Cavernous hemangioma |
| 3 | female | 62 | | Oligodendroglioma |
| 4 | male | 75 | | Glioblastoma |
| 5 | male | 20 | | Cavernous hemangioma |
| 6 | female | 27 | | Lateral ventricle glioma |

Control brain tissues were obtained from 6 patients undergoing cortical resection for glioma or cavernous hemangioma. These tissues were located at least 2 cm away from the lesion, and only samples confirmed to be histologically normal were included.

**Table S3. Basic characteristics of volunteers**

| Volunteer Number | Sex | Age | Lactate Content (mmol/L) |
| --- | --- | --- | --- |
| 1 | female | 22 | 0.403394167 |
| 2 | male | 32 | 0.295515833 |
| 3 | female | 27 | 0.148763333 |
| 4 | male | 31 | 1.921945 |
| 5 | female | 24 | 0.8023475 |
| 6 | male | 32 | 1.234345 |
| 7 | female | 21 | 0.821785 |
| 8 | male | 29 | 1.616776667 |
| 9 | male | 28 | 2.09834 |
| 10 | female | 32 | 2.360745 |

**Table S4. IP-MS analysis of anti-FH**

**Table S5. Lactylation scoring gene set**
